# Benchmarking of Whole Exome Sequencing and *Ad Hoc* Designed Panels for Genetic Testing of Hereditary Cancer

**DOI:** 10.1038/srep37984

**Published:** 2017-01-04

**Authors:** Lídia Feliubadaló, Raúl Tonda, Mireia Gausachs, Jean-Rémi Trotta, Elisabeth Castellanos, Adriana López-Doriga, Àlex Teulé, Eva Tornero, Jesús del Valle, Bernat Gel, Marta Gut, Marta Pineda, Sara González, Mireia Menéndez, Matilde Navarro, Gabriel Capellá, Ivo Gut, Eduard Serra, Joan Brunet, Sergi Beltran, Conxi Lázaro

**Affiliations:** 1 Hereditary Cancer Program, Joint Program on Hereditary Cancer, Catalan Institute of Oncology, IDIBELL campus in Hospitalet de Llobregat, Catalonia, Spain; 2 Centro Nacional de Análisis Genómico (CNAG-CRG), Center for Genomic Regulation, Barcelona Institute of Science and Technology (BIST), Barcelona, Catalonia, Spain; 3 Universitat Pompeu Fabra (UPF), Barcelona, Catalonia, Spain; 4 Genetic Variation in Cancer Group, Joint Program on Hereditary Cancer, Institut de Medicina Predictiva i Personalitzada del Càncer, Badalona, Catalonia, Spain; 5 Hereditary Cancer Program, Joint Program on Hereditary Cancer, Catalan Institute of Oncology, IdibGi in Girona, Catalonia, Spain

## Abstract

Next generation sequencing panels have been developed for hereditary cancer, although there is some debate about their cost-effectiveness compared to exome sequencing. The performance of two panels is compared to exome sequencing. Twenty-four patients were selected: ten with identified mutations (control set) and fourteen suspicious of hereditary cancer but with no mutation (discovery set). TruSight Cancer (94 genes) and a custom panel (122 genes) were assessed alongside exome sequencing. Eighty-three genes were targeted by the two panels and exome sequencing. More than 99% of bases had a read depth of over 30x in the panels, whereas exome sequencing covered 94%. Variant calling with standard settings identified the 10 mutations in the control set, with the exception of *MSH6* c.255dupC using TruSight Cancer. In the discovery set, 240 unique non-silent coding and canonic splice-site variants were identified in the panel genes, 7 of them putatively pathogenic (in *ATM*, *BARD1*, *CHEK2*, *ERCC3*, *FANCL*, *FANCM*, *MSH2*). The three approaches identified a similar number of variants in the shared genes. Exomes were more expensive than panels but provided additional data. In terms of cost and depth, panels are a suitable option for genetic diagnostics, although exomes also identify variants in non-targeted genes.

Hereditary cancer accounts for about 3% of all cancers (reviewed by Rahman)^1^,^2^ and is caused by inherited or *de novo* germline mutations in high-penetrance predisposition cancer genes. Approximately 100 cancer predisposition genes have been described in the literature^1^,^2^. The presence of a mutation in one of these genes predisposes to certain types of tumors with varying penetrance, with a lifetime cancer risk as high as 80% in patients with mutations in *BRCA1* or *BRCA2*^3^. Genetic testing for hereditary cancers has become a paradigm of personalized or precision medicine in the field of cancer^4^,^5^,^6^, helping to define the risk of each family member and enabling more effective family surveillance, management and follow-up^7^. In addition, carriers can now benefit from specific surgical and chemotherapeutic treatment strategies^8^,^9^.

The introduction of next-generation sequencing (NGS) for routine analysis has changed the way genetic testing is conceived and delivered^10^,^11^,^12^. Sanger sequencing was considered the gold standard for genetic diagnostics but it is a laborious and expensive procedure. Now, several dozen genes can be sequenced in a similar time frame and at a comparable cost to the Sanger analysis of only one large single gene^13^,^14^. As such, the inclusion of high- and moderate-risk genes in a diagnostic sequencing panel may provide additional relevant clinical information for families^15^,^16^,^17^,^18^. However, it is crucial to apply the ACCE model (Analytic validity, Clinical validity, Clinical utility and Associated ethical, legal and social implications) when implementing a new genetic test for diagnostic purposes, irrespective of the technical potential and cost advantages of the new test^19^.

Gene panels have been proposed as a cost-effective tool to address the underlying genetic causes of genetically heterogeneous disorders. Whole exome sequencing, by contrast, generates information for all known genes in the genome that can be used through the life of the individual and may prove more cost-effective in the long term, especially in the context of universal health systems^20^,^21^,^22^.

Here we present a benchmarking study in which 24 samples were NGS sequenced using three different approaches for library construction (two hereditary cancer panels and a whole exome panel). Our main goal is to assess the relative advantages and disadvantages of each methodology for diagnostic purposes.

## Results

**Figure 1:**
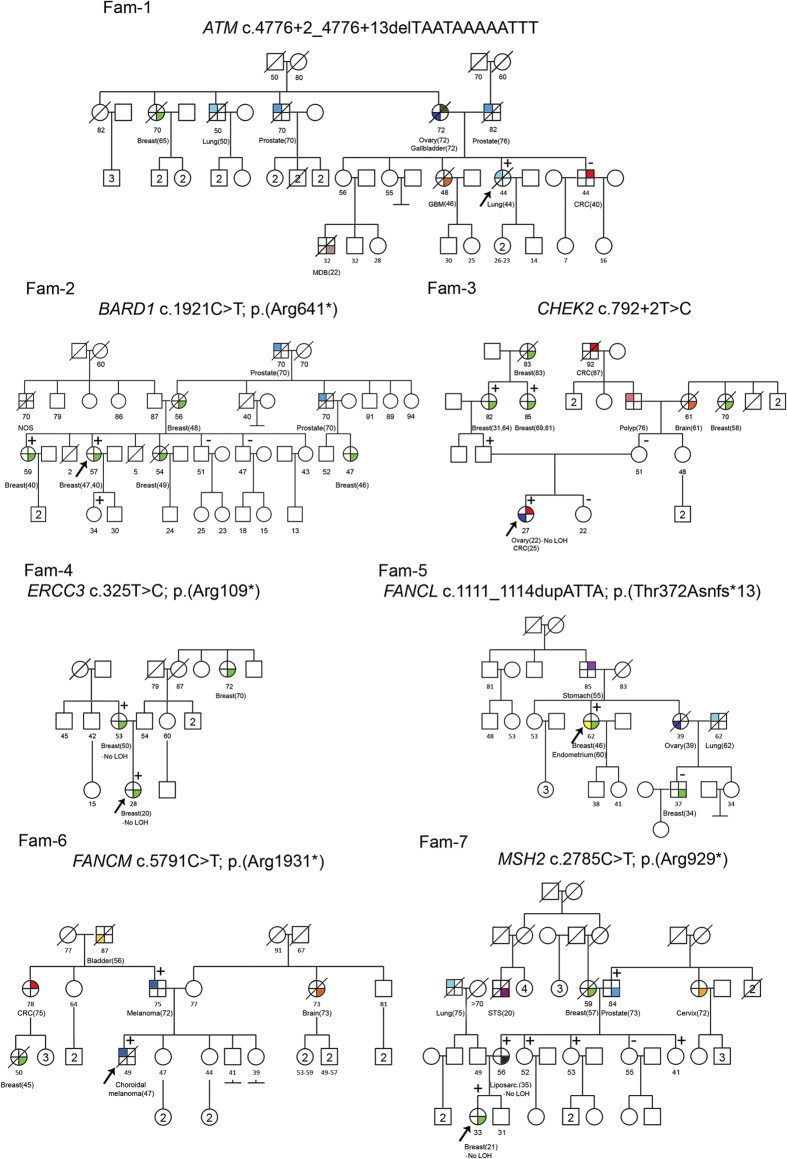
Pedigrees of the families in which a putative pathogenic mutation was identified in the panel genes. Filled quarters of symbols indicate patients affected by cancer (each color refers to a specific type). Current age, age at death and age at diagnosis (in brackets), when available, are also detailed. Putative pathogenic mutations are shown at the top of each pedigree; proband is marked by an arrow, carrier status was studied in available relatives, and those carrying/not carrying the variant are marked with +/− respectively. CRC, colorectal cancer; GBM; glioblastoma; Liposarc., Liposarcoma; LOH, loss of heterozygosity; MDB; medulloblastoma; NOS, not otherwise specified cancer; STS, soft tissue sarcoma.

We analyzed 24 affected individuals with suspicion of hereditary cancer (Table 1, Fig. 1 and Fig. S1). Ten carriers of known pathogenic mutations challenging for NGS formed a control set; fourteen additional samples were sequenced as a discovery set. Three approaches were used, based on distinct gene libraries: (i) a Custom Hereditary Cancer Panel (**I2HCP**, **I**CO-**I**MPPC **H**ereditary **C**ancer **P**anel, SureSelectXT Custom 3–5.9 Mb, Agilent Technologies) (Santa Clara, California) (Castellanos, *et al*., unpublished data); (ii) the TruSight Cancer Sequencing Panel (**TSCP**, Illumina) (San Diego, California); and (iii) the SureSelect Human All Exon v5 (**WES**, Agilent) (Table 2). **I2HCP** is an in-house customized panel of 122 genes designed to cover the coding exons and intron-exon boundaries of genes associated with moderate or high risk of hereditary cancer. **TSCP** targets 94 hereditary cancer genes, 83 of which are in common with the **I2HCP** (Table S1). The SureSelect **WES** kit was used to target all human coding exons, including those of the hereditary cancer genes covered by the panels.

### Run and Mapping Quality

Quality control summary data indicated that the total number of passing filter reads was very similar between the two panels (approx. 7.5 million reads) and approximately 9 times higher in the **WES** (Table S2). The percentage of unique-mapping reads was higher in the **WES** (94.77%) than in **I2HCP** and **TSCP** (90.28% and 87.77%, respectively). The percentage of duplicate reads was much higher in **TSCP** (66.16%), using tagmentation and a small amount of starting DNA, than in **I2HCP** (2.76%) and **WES** (7.33%).

### Target Regions, Read Depth and Coverage

**Figure 2:**
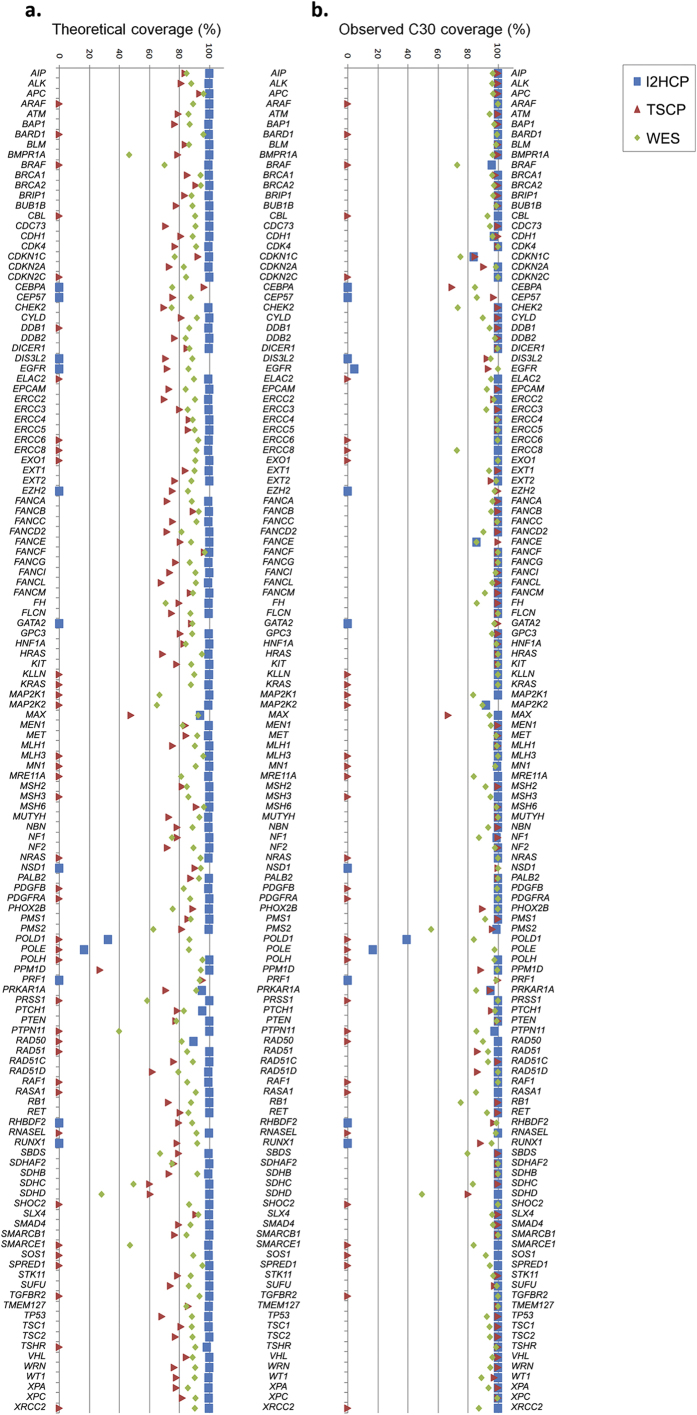
Theoretical and observed coverage of the 132-gene Diagnostic Region of Interest (DxROI): base percentage of the DxROI of the 132 genes targeted by any of the panels and the exome, covered by the three different sequencing strategies. (**a**) *Theoretical coverage.* Percentage coverage of the DxROI for each gene is obtained by comparing the designed target regions, as provided by each manufacturer (TSCP and WES) or aimed for in the I2HCP design. (**b**) *Observed coverage*. Percentage of DxROI bases of each gene effectively covered at a read depth ≥30x (C30) by each strategy; the median of the 24 samples is shown.

The Diagnostic Region Of Interest (DxROI) for a given set of genes is defined here as the sum of the fragments defined by the coding bases of the coding exons plus 20 bp (either intronic, 5′-UTR or 3′-UTR) surrounding each of them. The target regions were compared among themselves but also to the DxROI (Table S3). The reference sequence is the consensus coding sequence (CCDS)^23^, which contains protein-coding sequences with high-quality annotations. Depending on the purpose of the analysis, we evaluated the set of 83 genes common to both panels and the exome (Table S1), or the set of common genes plus the 49 genes with *CCDS ID* included in either of the panels (and the exome), totaling 132 genes. Figure 2 and Table S4 show the percentage of the 132-gene DxROI encompassed by each of the supplied target regions for each gene.

In hereditary cancer, where germline mutations are expected, 30 reads per base is considered the minimum coverage (hereafter, C30) for high-sensitivity heterozygote detection^24^,^25^. Average mean read depths of 497x, 455x and 129x were obtained for the 83-gene DxROI by **I2HCP**, **TSCP** and **WES**, respectively. More than 99% of targeted bases were covered at C30 by both panels, while **WES** was slightly less effective (94% on average) (Fig. S2). The C10 base percentage is also plotted, showing the putative potential of **WES** if more reads per sample were obtained or if a Whole Exome capture kit were used that favors the medically relevant genes.

**Figure 3:**
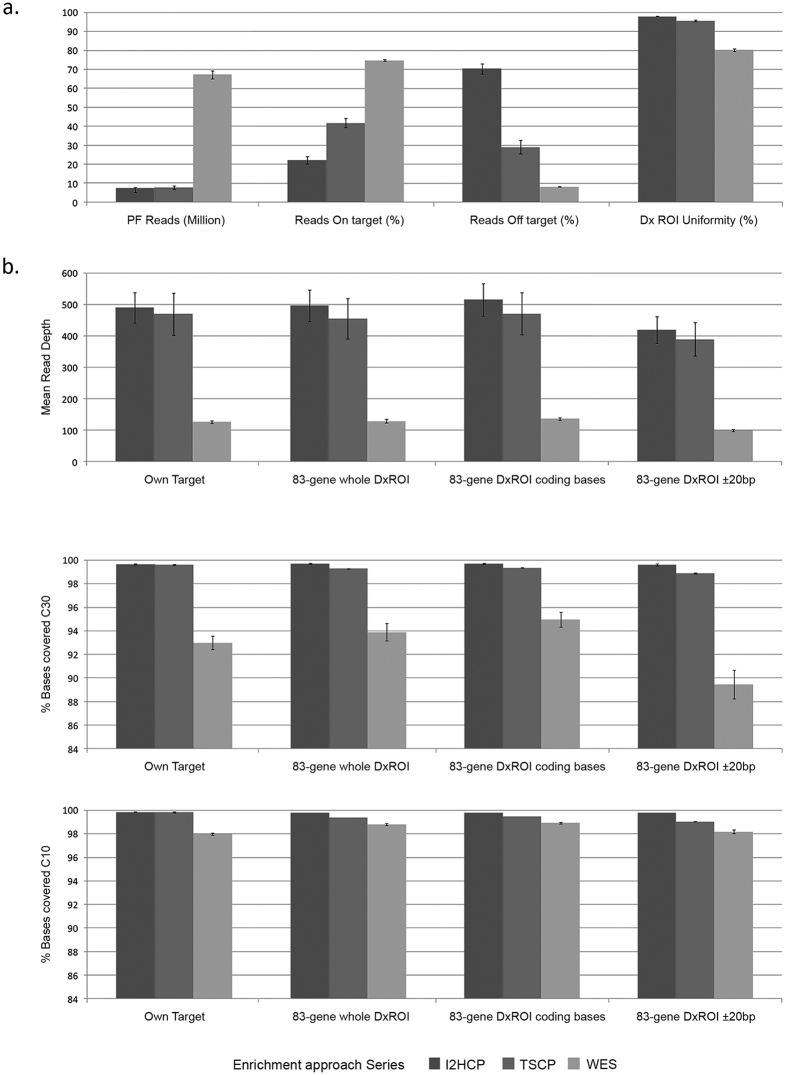
Comparison of main coverage metrics. Average of all samples and 95% confidence interval are shown in each bar plot for the three approaches. (**a**) Performance metrics: passing filter (PF) reads; percentage of on-target reads, defined as any read overlapping at least one base the target region defined by the corresponding approach, versus total PF reads; percentage of off-target reads, defined as those within regions more than ±500-bp outside the designed own target regions; and uniformity of the coverage of the 83-gene DxROI (Diagnostic Region of Interest), calculated as the fraction of CCDS coding exons plus 20 bp boundaries reaching a mean read depth within ±70% of the mean read depth over all coding exons plus 20 bp boundaries. (**b**) Mean read depth, percentage of bases with read depth at least 30x and 10x versus: own target regions, the whole 83-gene DxROI, or considering the coding bases and their 20-bp boundaries separately.

The performance of the three approaches was further compared by considering the percentage of on-target and off-target reads (*versus* own targets), the coverage uniformity of the 83-gene DxROI, and the mean read depth. The percentage of C30 and C10 bases versus the whole 83-gene Dx ROI or the 83-gene DxROI broken down into coding bases and 20 bp of intronic/UTR surrounding bases, were also considered (Fig. 3). **I2HCP** and **TSCP** reached >99% C30 on their own target regions, whole DxROI and DxROI coding bases. **TSCP**, but not **I2HCP**, dropped slightly below 99% C30 in the intronic and UTR bases of the DxROI. **WES** had the highest on-target (75%) and the lowest off-target (8%) percentages, and although the mean coverage to which it was sequenced was around 3.5 times less than **I2HCP** and **TSCP**, the C30 was >94% on the 83-gene DxROI coding bases and >89% on the 20 bp surrounding the coding bases. According to diagnostic quality standards^25^, all regions not reaching the required C30 must be Sanger sequenced; **WES** yielded an average of 240 fragments per sample to be Sanger sequenced for the whole set of 83 common genes, whereas **I2HCP** and **TSCP** yielded 9 and 19, respectively (Fig. S3). Enrichment efficiency versus GC content was also evaluated, with different patterns observed between capture methods (Fig. S4).

### Variant Detection

An average of 111 variants per sample (range 85–119) were found in the coding regions plus two intronic surrounding bases (canonical splice sites) from the common genes (Fig. S5). Average concordance was high: 93.8% between **I2HCP** and **TSCP**, 92.1% between **I2HCP** and **WES**, and 93.2% between **TSCP** and **WES**. On the whole, false positives and false negatives were fairly uniformly distributed among the three approaches. They were mostly linked to a small number of reads and attributable to variant calling (data not shown).

#### Variant Detection in the Control Set

Variant calling with standard settings identified the 10 pathogenic mutations in the control set with the three approaches, with the exception of the *MSH6* mutation c.255dupC in **TSCP** (Table 1). This mutation was probably not called due to a lower variant read ratio (0.32 in **TSCP** *vs* 0.43 in **I2HCP** and 0.45 in **WES**) and the lack of forward reads at the end of that GC-rich exon. However, SAMtools called this variant when the p-value threshold parameter was changed from 0.5 to 0.75.

#### Variant Detection in the Discovery Set

A total of 240 unique non-silent coding and canonic splice-site variants were identified in the 132 panel genes for the 14 samples in the discovery set. Seven could be classified as highly probable deleterious mutations in *ATM*, *BARD1*, *CHEK2*, *ERCC3*, *FANCL*, *FANCM* and *MSH2* genes (Table 1), corresponding to frameshift, nonsense or canonic splice site mutations. All of these mutations were confirmed by Sanger sequencing and detected by the three platforms, except for a nonsense mutation in *BARD1* that was not identified by the **TSCP**, which does not include this gene. It should be noted that the putative pathogenic mutations detected, with the exception of the *ATM* splice-site variant, have previously been reported as associated with cancer (see references in Table S5). Further investigation of the *ATM* and *CHEK2* variants at the canonical splice site by RNA analysis revealed altered splicing (Fig. S6A and B).

To support the hypothesis that these variants could be responsible for the observed phenotype, cosegregation studies were performed in each family, when possible, in addition to LOH of the variant in the available tumor samples (Fig. 1). While the clinical information for the families was good, a limited number of DNA samples from other affected relatives were available. Three of the identified mutations showed cosegregation with cancer (*CHEK2*, *ERCC3* and *FANCM*). *MSH2* mutation was present in all available affected relatives and also in two unaffected relatives. No LOH was evident in the three tumor samples available for analysis.

In the panel genes we identified 43 additional missense variants or in-frame insertions or deletions with a population frequency lower than 1% (Table S6). Their effect on the protein is not as straightforward as those deduced for truncating and canonical splice site mutations.

Finally, as expected, **WES** detected numerous variants with predicted high or moderate effect in several genes not included in the panels (Table S7). A multidisciplinary group composed of clinical geneticists, molecular biologists and NGS specialists evaluated the filtered list according to patient/family phenotype and compiled a shortlist of 24 putatively relevant variants in genes previously unrelated to hereditary cancer (Table S8). Fourteen of these variants were detected in six of the seven families that were negative for highly probable deleterious mutations in the panel genes. Interestingly, in two of these cases variants of genes involved in genome (*POLQ*) or chromatin (*SETD4*) stability were detected, so these findings also open new avenues for investigating cancer susceptibility.

### Comparison of approaches and Turn-Around Times

A study was performed of the consumables, computing, data storage and time requirements for the overall sequencing and analysis process to establish a rough comparison of the sequencing options and turn-around time for the three approaches (Table 2). The overall price of consumables for DNA capture and library preparation was similar for the three libraries (around €150–200/sample). Library preparation time is similar in **I2HCP** and **WES** (4–5 days) and slightly shorter in **TSCP** (3 days). The price of sequencing and run-time per sample depend on the specific sequencing options (Table 2). Crucially, WES requires around 10 times more sequences. In terms of data storage, panel results generate an average of 2 GB of data per sample, whereas **WES** produces about 20 GB per sample. Although computing (CPU) time to obtain alignments and variant lists is approximately double for **WES** (Table 2), parallelization means that results can be obtained in only a few hours (wall clock time) in both cases.

## Discussion

This study reports a comprehensive comparison of *ad hoc* panels for hereditary cancer syndromes and **WES** in the clinical diagnostic setting, where high standards of quality and accuracy are required, and laboratories are under pressure to deliver short turn-around times for multiple tests.

From the clinical perspective, **I2HCP** and **WES** proved robust in the detection of *bona fide* previously identified pathogenic point mutations. The commercial **TSCP** panel missed one mutation at the end of a GC rich exon. Since the design and protocols of standard commercial kits are fixed, there is not much that can be done to improve coverage of certain regions from the user perspective. Therefore, regions of interest with bad quality or poor coverage need to be analyzed with alternative techniques. Notably, all platforms identified putative pathogenic mutations in half of the discovery families tested, either in high-risk genes (such as *MSH2*), in moderate-risk genes (*CHEK2*, *ATM*, *BARD1*) or in genes related to hereditary cancer susceptibility without a well-known associated risk (*FANCM*, *FANCL*, *ERCC3*). Co-segregation (*CHEK2*, *ERCC3* and *FANCM*) and functional effect at RNA level (*ATM*, *CHEK2*) have been demonstrated in some genealogies, although large case-control sequencing studies and larger family-based studies will be needed to better define the risks before most of these genes can be routinely used in the clinical setting. **WES** identified 24 potentially damaging mutations in the 14 probands. The value of these findings is difficult to estimate, but they can put and end to the diagnostic odyssey and can also shed light on the compound deleterious effects of multiple mutations.

High performance standards are essential in the clinical diagnostic setting^12^,^21^,^25^,^26^,^27^,^28^. The critical difference between panel sequencing and **WES** is coverage. Under standard conditions, **WES** aims for a mean read depth of around 100×^29^,^30^, while panel sequencing generally targets a range of 200–1000×^14^,^27^,^31^. Assuming a “standard run” for both approaches, our study clearly demonstrates that the number of target region bases not reaching C30 is much higher in **WES** than in panel sequencing. As such, the number of Sanger sequences required for diagnostic-quality results increases for **WES**, thereby increasing the overall cost of the test. It is obviously cheaper to sequence a panel than an exome due to the much smaller capture region, but this gap is narrowing as, for example, the sequencing costs of an exome are 50% lower with a HiSeq 4000 compared to a HiSeq 2000. Moreover, the development of comprehensive panels targeting most clinically relevant genes (around four thousand), such as TruSight One (Illumina), or exome kits offering better coverage for disease-associated targets (such as, Agilent’s SureSelect Clinical Research Exome and SureSelect Focused Exome or Roche’s SeqCap EZ MedExome) would be more cost-effective than standard **WES**. However, it must be taken into consideration that our **I2HCP** panel required a cost for development and validation that has not been included here and that **TSCP** and **WES** are both labeled for research use only, meaning that proper validation by the user’s laboratory is required before they can be applied in a diagnostic setting.

On the other hand, panel data results are easier to interpret than full **WES** results, since fewer genes are considered and we know more about their mutational spectrum and clinical relevance in cancer. However, in favor of **WES**, a two-step approach could be used in bioinformatic analysis by first identifying putative pathogenic variants in a list of candidate genes before investigating the sequence of the other exome genes obtained in the same experiment.

In this study we have focused on the comparison of single nucleotide variant (SNV) and insertion/deletion detection, since these approaches are highly sensitive and specific to detect this kind of mutations. However, other mutations such as copy number variations (CNV) are difficult to detect with these methods. Therefore, our diagnostics workflow includes MLPA (Multiplex Ligation-dependent Probe Amplification) of the main genes of clinical interest. Alternative techniques such as aCGH (array comparative genomic hybridization), with higher throughput but lower resolution, are other viable options. In the future, additional tests might not be necessary if new targeting approaches, like the Agilent OneSeq 1 Mb CNV Backbone + Custom Panel, or bioinformatic methods overcome the current limitations.

Turn-around time is important in the clinical setting^25^,^32^. For several cancer types, results must be obtained quickly because chemotherapy (*i.e. BRCA* status and PARP inhibitors) or surgery decisions depend on mutational status^8^. Shorter turn-around time is achieved with panel results, as the analysis itself is quicker and the list of variants to analyze/validate is much shorter. However, a partial analysis of the exome could also be performed, which would put it on a par. Sequencer type and run times are also important considerations: panels can be sequenced in a benchtop NGS sequencer such as MiSeq (Illumina), whereas **WES** needs a higher sequencing throughput as provided by a HiSeq 2000 or a newer sequencer, which is not always feasible for clinical laboratories (Table 2). However, sequencing cost per GB is higher for MiSeq than for HiSeq.

The clinical utility of a given test is critical when choosing a methodology. Ethical issues related to putative incidental findings should be considered and discussed with the patient before ordering a genetic test^10^,^33^,^34^. Although considerable advances have been made over the last decade in our understanding of the molecular basis of several cancer syndromes, the translation of this knowledge into clinical surveillance and patient management remains limited. The risks associated with mutations in several cancer genes are still unknown, making it difficult to apply the genetic information obtained in daily patient care. As **WES** reveals mutations in uncharted genes, a degree of uncertainty remains that must be addressed.

There are benefits of analyzing target genes using *ad hoc* panels, as they focus on the regions of greatest interest and return clinical-grade results at a good cost. However, as our knowledge grows frequent updates are necessary. WES, by contrast, identifies the variants of interest, although some regions do not reach clinical sensitivity; information about other clinically relevant observations is immediately available and hardly any updates will be necessary.

## Methods

### Patients and DNA Extraction

A total of 24 affected individuals were selected from unrelated families with family history of cancer attending our Cancer Genetic Counseling Units. Genetic testing had been indicated because of: 1) early-onset cancer; and/or 2) multiple tumors in a patient, and/or 3) familial aggregation of cancer suggestive of a dominant pattern of inheritance (Table 1, Fig. 1 and Fig. S1). Ten cases were positive for technically challenging mutations (control set). The remaining 14 were negative in the routine analysis of one or a few candidate genes by conventional Sanger sequencing (discovery set). Informed consent was obtained from all subjects and the study received the approval of the Ethics Committee of the Institut d’Investigació Biomedica de Bellvitge (IDIBELL) (PR073/12), which fulfills the International ethical guidelines for biomedical research involving human subjects. The methods were carried out in accordance with the approved guidelines. Genomic DNA from peripheral blood was used for NGS.

### Next-generation Sequencing: Library Preparation

Library preparation was conducted according to the manufacturers’ instructions (**TSCP** at the Catalan Institute of Oncology (ICO), **WES** and **I2HCP** at the National Center for Genomic Analysis (CNAG)).

#### SureSelect Custom Panel (I2HCP)

A total of 122 genes were targeted for a final capture size of 400 kb (SureSelectXT Custom 3–5.9 Mb, Agilent) (Castellanos, *et al*., unpublished data), according to Agilent’s SureSelect protocol for Illumina paired-end sequencing. Briefly, 3.0 μg of genomic DNA was sheared on a Covaris E210 instrument (Covaris, Woburn, Massachusetts). The fragment size (150–300 bp) and the quantity were confirmed with an Agilent 2100 Bioanalyzer 7500 chip. Fragmented DNA was end-repaired, adenylated and ligated to Agilent indexing-specific paired-end adaptors. The DNA with adaptor-modified ends was PCR amplified (6 cycles, Herculase II fusion DNA polymerase, Agilent) with SureSelect Primer and SureSelect Pre-capture Reverse PCR Primer, quality controlled on the DNA 7500 assay for the library size range of 250 to 450 bp and hybridized for 24 hours at 65 °C (Applied Biosystems 2720 Thermal Cycler). The hybridization mix was washed in the presence of magnetic beads (Dynabeads MyOne Streptavidin T1, Life Technologies, Carlsbad, California) and the eluate was PCR amplified (16 cycles) in order to add the index tags using SureSelectXT Indexes for Illumina. The final library size and concentration were determined on an Agilent 2100 Bioanalyzer 7500 chip.

#### TruSight Cancer Panel (TSCP) (Illumina)

This enrichment system targeted 94 genes associated with hereditary cancer syndromes. Libraries were generated using TruSight Rapid Capture along with the TruSight Cancer Sequencing Panel (Illumina), according to the manufacturer’s sample preparation protocol. Briefly, 50 ng of each DNA sample were enzymatically fragmented and adapter sequences were added to the ends. The fragmented DNA was purified and barcodes and common adapters required for cluster generation and sequencing were PCR-added. After cleanup, 500 ng of each 12 DNA libraries were pooled. Then the libraries were hybridized twice to specific capture probes; the unhybridized material was washed away and the captured fragments were amplified using PCR followed by purification. The enriched libraries were quantified using a Qubit 2.0 Fluorometer and their quality was evaluated using a Bioanalyzer 2100 and the High Sensitivity DNA Kit (Agilent Technologies). Libraries were diluted and pooled to obtain the final sequencing equimolar pool.

#### Whole Exome Sequencing (WES)

Library preparation for the capture of selected DNA regions (Agilent Human All Exon 50 Mb v5, Agilent Technologies) was performed with the same protocol as for **I2HCP** (see above).

### Next-generation Sequencing: Run

Each **WES** library was sequenced at CNAG on an Illumina HiSeq 2000 system in a fraction of a sequencing lane, following the manufacturer’s protocol, with a paired end run of 2 × 101 bp, with at least 98% of the target region covered at C10, following CNAG standards for exome sequencing. For **TCSP** and **I2HCP** panels, 24 samples were sequenced on one lane of an Illumina HiSeq 2000 with 2 × 101 bp paired-end mode. In all cases, image analysis, base calling and quality scoring were processed using the manufacturer’s Real-Time Analysis software (RTA 1.13.48, HCS 1.5.15.1), followed by generation of FASTQ sequence files in CASAVA.

### Next-generation Sequencing: Data Analysis and Variant Calling and Prioritization

The same algorithms and settings were used for all three approaches. Reads were hard-trimmed from the end up to the first base, with a quality of at least 10. Reads at least 40 nt in length were mapped to the human genome build hg19 [hs37d5] (http://www.1000genomes.org/category/reference) using GEM toolkit^35^ allowing up to 4 mismatches. Alignment (.bam) files containing only properly paired, unique-mapping reads were processed using Picard tools release 1.110 (http://picard.sourceforge.net) to add read groups and to remove duplicates. The Genome Analysis Tool Kit (GATK) version 3.1 (https://www.broadinstitute.org/gatk/)^36^ was used for local realignment. Processed.bam files were submitted to variant calling for single nucleotide variants and small insertions and deletions using SAMtools version 0.1.19 (http://samtools.sourceforge.net/)^37^. Functional annotations were added using snpEff (http://snpeff.sourceforge.net/)^38^ with the GRCh37.75 database. Human dbSNP version 137, population frequencies from 1000 Genomes and the Exome Variant Server, as well as conservation and deleteriousness predictions from dbNSFP (http://sites.google.com/site/jpopgen/dbNSFP)^39^, were annotated using snpSift (http://snpeff.sourceforge.net/SnpSift.html)^40^.

Given the number of variants identified in **WES**, and in order to prioritize them, variants were filtered according to several stringent criteria (Table S8). Briefly, we discarded variants i) with an allelic population frequency ≥1% according to the 1000 Genomes Project and Exome Variant Server data annotated with dbNSFP v2.5 (http://varianttools.sourceforge.net/Annotation/DbNSFP); ii) observed more than once in our set of 24 samples; iii) with low sequencing quality, or iv) with an allele ratio <0.25. Of the variants that met these criteria, we selected those most likely to be pathogenic according to the SnpEff v.3.6 annotation tool (http://snpeff.sourceforge.net), which classified them as HIGH impact (frameshift, nonsense, STOP lost/gain, exon/chromosome deletion, canonic splice-site), and directly related to cancer according to the VarElect prioritization tool (http://varelect.genecards.org).

### Variant validations, cosegregation and LOH studies

Putative pathogenic variants confirmation, cosegregation studies and loss of heterezygosity analysis were performed by Sanger sequencing using the primers listed in Table S9.

### Cell cultures for RNA analysis

Human lymphocytes from fresh and frozen samples were maintained in RPMI 1640 medium (Invitrogen, Carlsbad, CA) supplemented with with PHA (Phytohemagglutinin) (10 μg/ml RPMI medium), 10% fetal calf serum and 1% penicillin-streptomycin (Sigma, Saint Quentin Favallier, France) in a 5% CO_2_ incubator at 37 °C. Human lymphocytes were cultured in the absence or presence of puromycin (Sigma Aldrich, St. Louis). Puromycin, a translational inhibitor that prevents potential degradation of unstable transcripts by the Nonsense-Mediated mRNA Decay (NMD) mechanism, was added to a final concentration of 100 μg/ml of culture for 4–6 hours prior to RNA isolation.

### RNA isolation and RT-PCR

Total RNA was isolated using TRIzol reagent (Invitrogen), according to the manufacturer’s instructions. Five hundred ng of total RNA from each sample were reverse-transcribed (RT) in a 20 μl reaction, which also contained 1x reaction buffer, 0.5 mM dNTPs, 1x DTT, 2 ng random hexamers and 200 units of Superscript II reverse transcriptase (Invitrogen, Carlsbad, CA, USA). The reaction conditions were 5 min at 65 °C, 10 min at 25 °C, 50 min at 42 °C, and 10 min at 70 °C. The subsequent PCR was performed using 2 μl of the cDNA mixture with specific primers targeting the region of interest (Table S9). RT-PCR products were separated by electrophoresis on a 1.5–3.0% agarose gel containing ethidium bromide and visualized by exposure to UV light and sequenced on an ABI 3730xl sequencer (Applied Biosystems, Foster City, CA) using Big Dye Terminator v3.1 cycle sequencing reaction kit (Applied Biosystems).

## Additional Information

**How to cite this article**: Feliubadaló, L. *et al*. Benchmarking of Whole Exome Sequencing and *Ad Hoc* Designed Panels for Genetic Testing of Hereditary Cancer. *Sci. Rep.* **7**, 37984; doi: 10.1038/srep37984 (2017).

**Publisher's note:** Springer Nature remains neutral with regard to jurisdictional claims in published maps and institutional affiliations.

## Supplementary Material

Supplementary Figures and Tables

Supplementary Table S7

## Figures and Tables

**Table 1:**
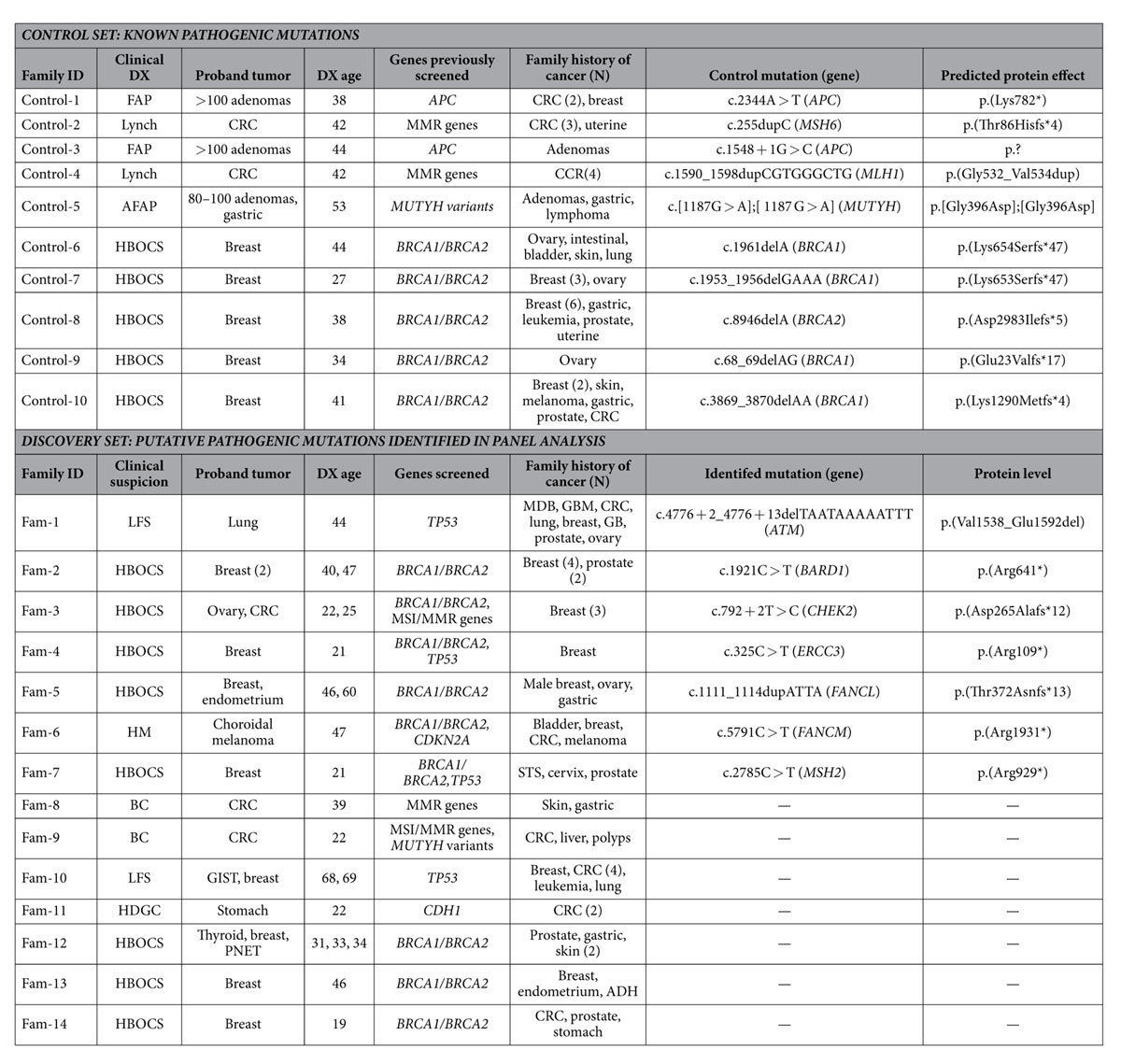
Patient description.

ADH, atypical ductal hyperplasia; AFAP, attenuated familial adenomatous polyposis; BC, Bethesda criteria; CRC, colorectal cancer; DX, diagnosis; FAP, familial adenomatous polyposis; GIST, gastrointestinal stromal tumors; GB, gallbladder; GBD; glioblastoma; HBOCS, hereditary breast and ovarian cancer syndrome; HM, hereditary melanoma; HDGC, hereditary diffuse gastric cancer; LFS, Li-Fraumeni syndrome; MDB; medulloblastoma; MMR, mismatch repair; MSI, microsatellite instability; PNET, pancreatic neuroendocrine tumor; STS, soft tissue sarcoma.

**Table 2:** Study design.

Enrichment approach		I2HCP	TSCP	WES
Library design	Kit, supplier	Custom SureSelect, Agilent	TruSight Cancer, Illumina	All Exon v5, Agilent
	Target region	400 Kb	255 Kb	50,400 Kb
	Target genes	122	94	≈21,500
	Target SNPs	47 identification, 43 risk	284 risk	All coding SNPs
Technical details	Input DNA	3 μg DNA	50 ng DNA	3 μg DNA
	Bait length	120-mer RNA	80-mer DNA	120-mer RNA
	Fragmentation	Covaris DNA shearing	Nextera tagmentation	Covaris DNA shearing
	Capture	In solution hybridization	In solution hybridization	In solution hybridization
	Prepared at	CNAG	ICO	CNAG
	Library prep. time	4–5 days	3 days	4–5 days
Run	Platform	Hiseq 2000, Illumina	Hiseq 2000, Illumina	Hiseq 2000, Illumina
	Kit	TruSeq SBS v3: 200 cycle	TruSeq SBS v3: 200 cycle	TruSeq SBS v3: 200 cycle
	Throughput	24 patients/lane, 1.5 Gb/patient	24 patients/lane, 1.5 Gb/patient	2.6 patients/lane, 13.6 Gb/patient
	Run time	11 day	11 day	11 day
Computing per sample	CPU time	50 h	44 h	93 h
	Storage	2.2 GB	1.7 GB	20.5 GB
Other sequencing options. HiSeq2500 rapid run	Kit	HiSeq rapid SBS kit v2, 200 cycle, 60 Gb	HiSeq rapid SBS kit v2, 200 cycle, 60 Gb	HiSeq rapid SBS kit v2, 200 cycle, 60 Gb
	Throughput	40 patients/flow cell	40 patients/flow cell	4.3 patients/flow cell
	Run time	27 h	27 h	27 h
Other sequencing options. MiSeq	Kit	MiSeq Reagent kit v3: 600 cycle, 15 Gb	MiSeq Reagent kit v3: 600 cycle, 15 Gb	Not feasible with MiSeq
	Throughput	10 patients/flow cell	10 patients/flow cell	
	Run time	39 h	39 h	
