# Supplementary material for: Benchmarking of Whole Exome Sequencing and Ad Hoc Designed Panels for Genetic Testing of Hereditary Cancer
Source: Sci Rep. 2017 Jan 4;7:37984. doi: 10.1038/srep37984 (PMC5209723; doi:10.1038/srep37984)
Supplement: Supplementary Figures and Tables [file srep37984-s1.doc]

**BENCHMARKING OF WHOLE EXOME SEQUENCING AND *AD HOC* DESIGNED PANELS FOR GENETIC TESTING OF HEREDITARY CANCER**

**Running Title:**

Hereditary cancer: benchmarking of panels and exome NGS

Lídia Feliubadaló, PhD^1^,*, Raúl Tonda, PhD^2,3^,*, Mireia Gausachs, PhD^1^,*, Jean-Rémi Trotta, BS^2,3^, Elisabeth Castellanos, PhD^4^, Adriana López-Doriga, BS^1^, Àlex Teulé, MD^1^, Eva Tornero, BS^1^, Jesús del Valle, PhD^1^, Bernat Gel, PhD^4^, Marta Gut, PhD^2,3^, Marta Pineda, PhD^1^, Sara González, BS^1^, Mireia Menéndez, PhD^1^, Matilde Navarro, MD^1^, Gabriel Capellá, MD, PhD^1^, Ivo Gut, PhD^2,3^, Eduard Serra, PhD^4^, Joan Brunet, MD, PhD ^5^, Sergi Beltran, PhD^2,3^ and Conxi Lázaro, PhD^1^

^1^Hereditary Cancer Program, Joint Program on Hereditary Cancer, Catalan Institute of Oncology, IDIBELL campus in Hospitalet de Llobregat, Catalonia, Spain; ^2^Centro Nacional de Análisis Genómico (CNAG-CRG), Center for Genomic Regulation, Barcelona Institute of Science and Technology (BIST), Barcelona, Catalonia, Spain; ^3^Universitat Pompeu Fabra (UPF), Barcelona, Catalonia, Spain; ^4^Genetic Variation in Cancer Group, Joint Program on Hereditary Cancer, Institut de Medicina Predictiva i Personalitzada del Càncer, Badalona, Catalonia, Spain; ^5^Hereditary Cancer Program, Joint Program on Hereditary Cancer, Catalan Institute of Oncology, IdibGi in Girona, Catalonia, Spain

*****The first three authors have equally contributed to this work.

**Correspondence to** Conxi Lázaro, PhD, Hereditary Cancer Program, Genetic Diagnosis Unit, Catalan Institute of Oncology (ICO-IDIBELL), Hospital Duran i Reynals, Gran Via 199-203, L’Hospitalet de Llobregat, 08908, Spain. Tel.: +34932607342; e-mail: [clazaro@iconcologia.net](mailto:clazaro@iconcologia.net).

**Figure S1. Pedigrees of the families without putative pathogenic mutations identified by gene panel analysis.** Shaded quarters of symbols indicate patients affected by cancer (each color refers to a specific type). Current age, age at death and age at diagnosis (in brackets), when available, are also detailed, proband is marked by an arrow. ADH, atypical ductal hyperplasia; CRC, colorectal cancer; GIST, gastrointestinal stromal tumors; NOS, not otherwise specified cancer; PNET, pancreatic neuroendocrine tumor; STS, soft tissue sarcoma.


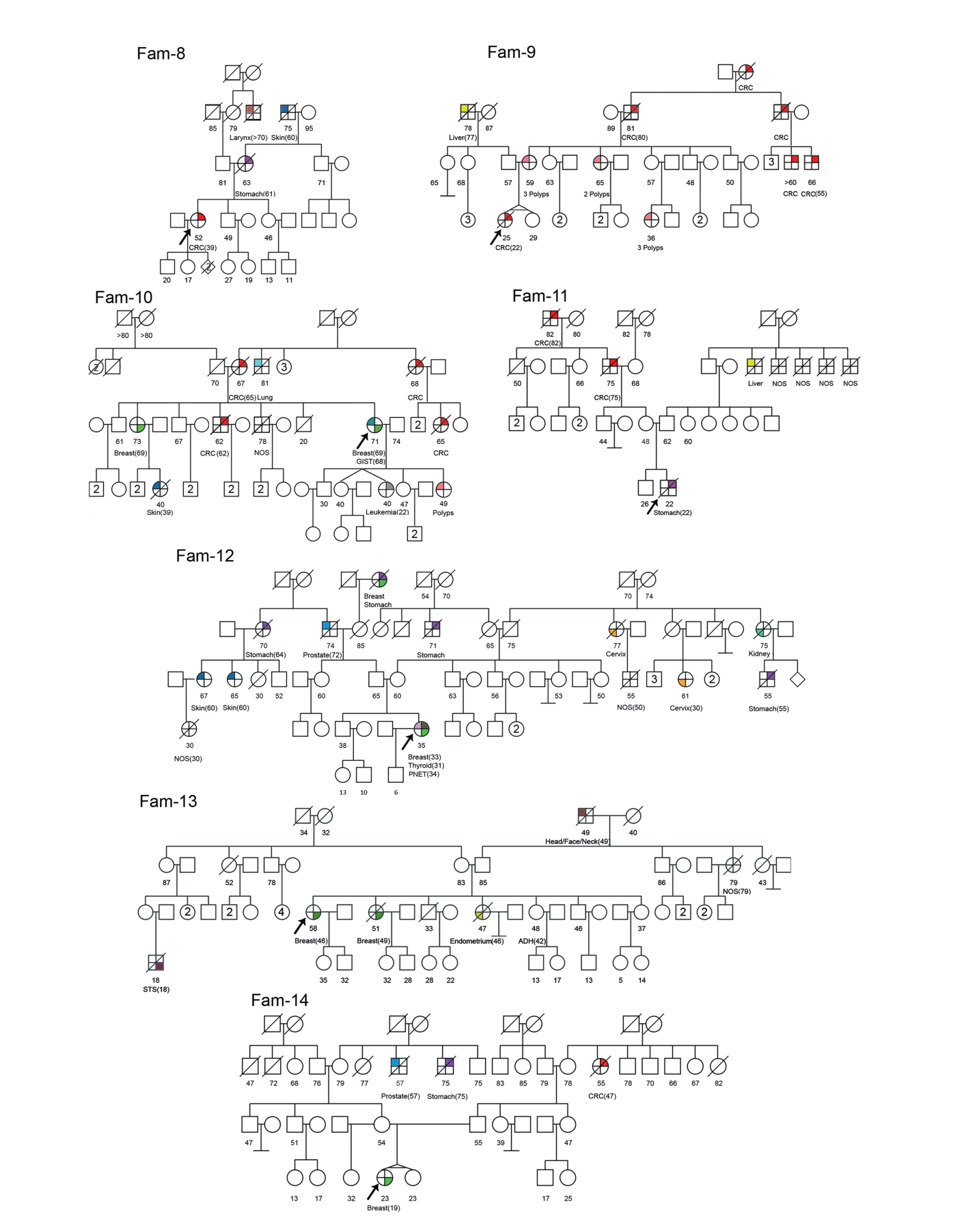


**Figure S2. C10 and C30 coverage percentage versus passing filter reads for each sample and approach.** Plots represent the percentage of bases with a minimum coverage of 30x (C30, red) and those with a minimum coverage of 10x (C10, blue) reached by each approach for each sample, against the total number of passing filter (PF) million reads, for the 83-gene Diagnostic Region of Interest.

**Figure S3. Number of Sanger sequences required for undercovered fragments of the 83 gene-Diagnostic Region of Interest (DxROI).** The figure illustrates the number of fragments of each sample (columns) and gene (rows) that should be Sanger sequenced. According to diagnostic standards all DxROI bases need to be covered with at least 30 reads. Regions that meet this condition are highlighted in green; other colors indicate the number of fragments that should be Sanger sequenced to complement the analysis of undercovered bases (represented as a color gradient).


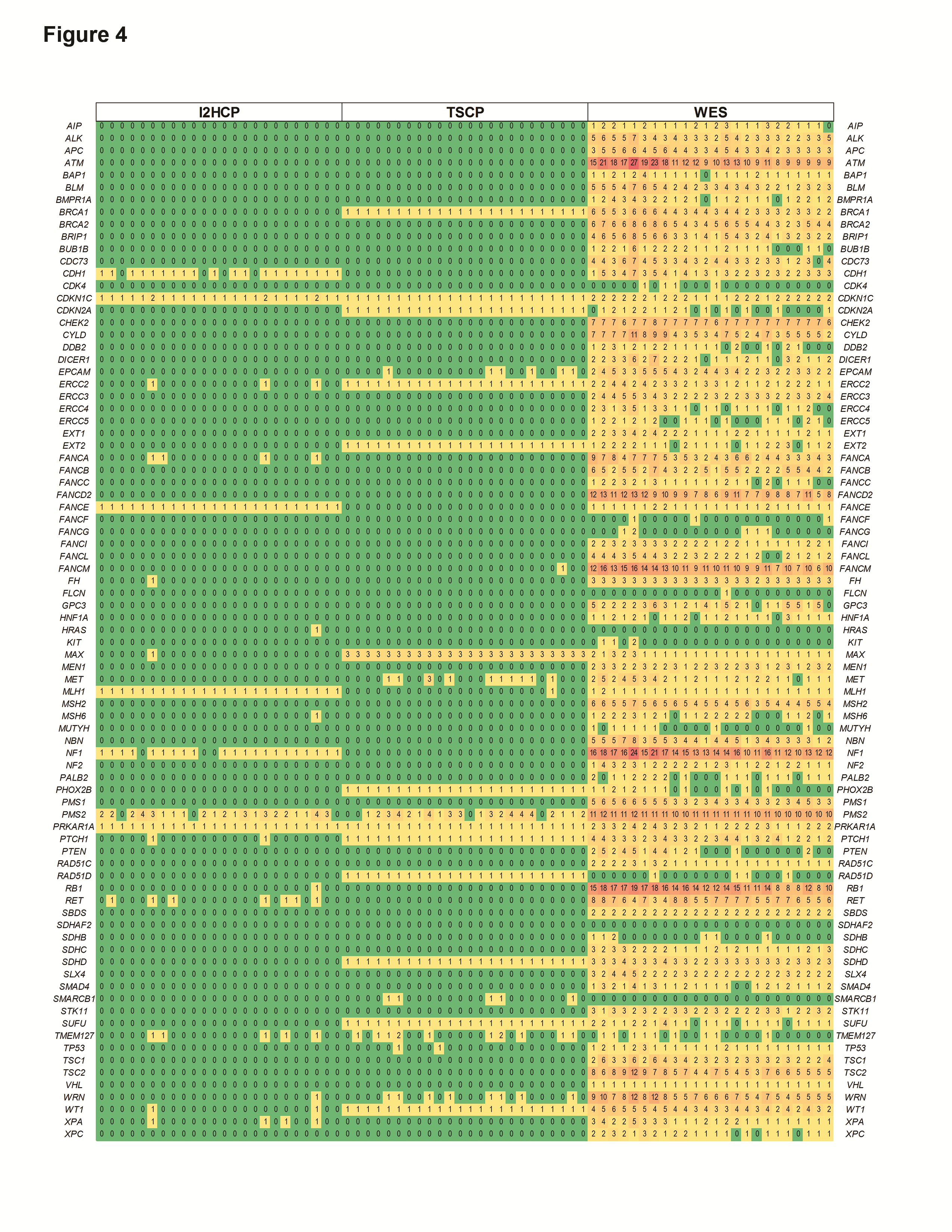


**Figure S4. Density plots showing sample mean read depth for both panels and WES versus GC content.** Sample average of the mean read depth of each 83-gene Diagnostic Region of Interest fragment (coding bases of coding exons plus 20bp boundaries), versus the GC percentage of that fragment.


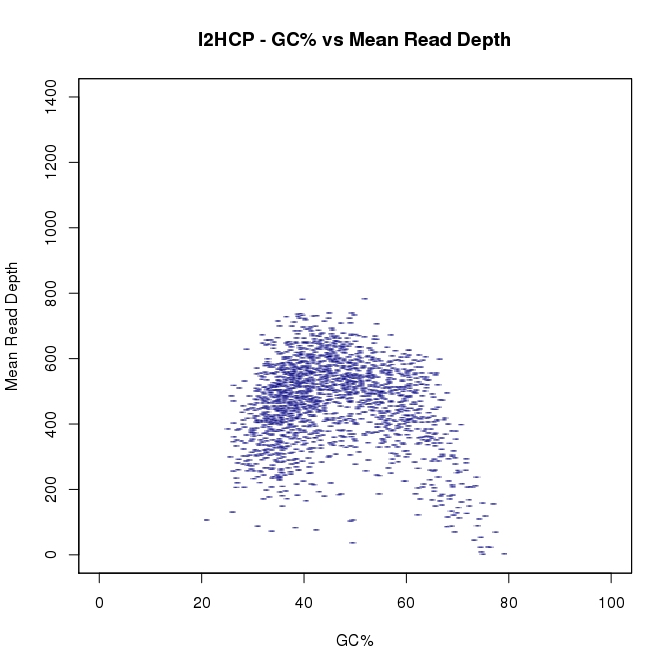

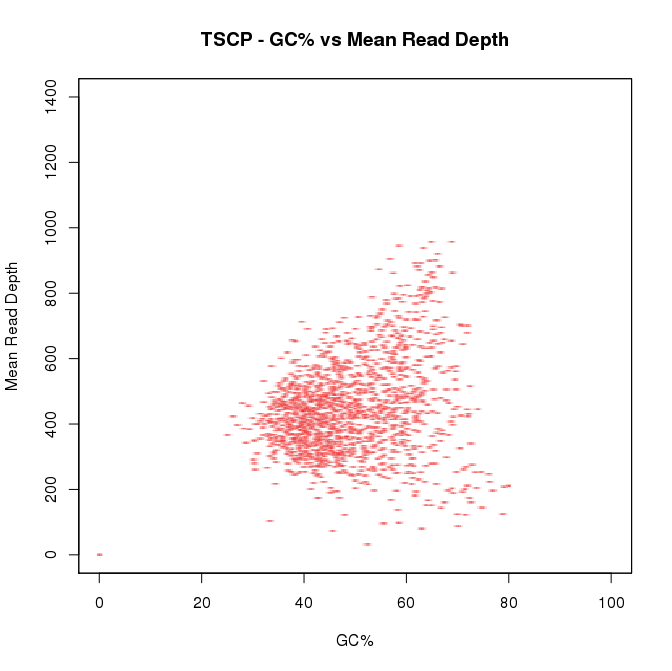

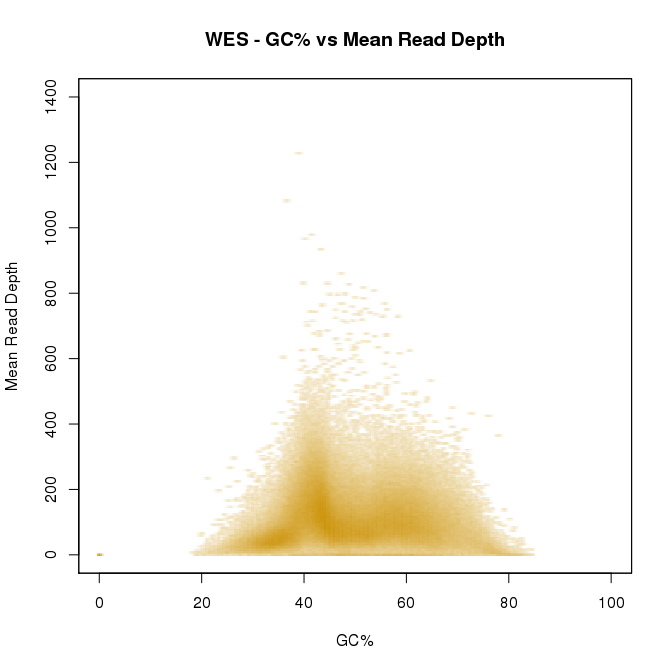


**Figure S5. Venn diagrams showing variant detection concordances and discordances between the three approaches in the 83 common genes (coding sequences ±2 bp) for all 24 samples.**


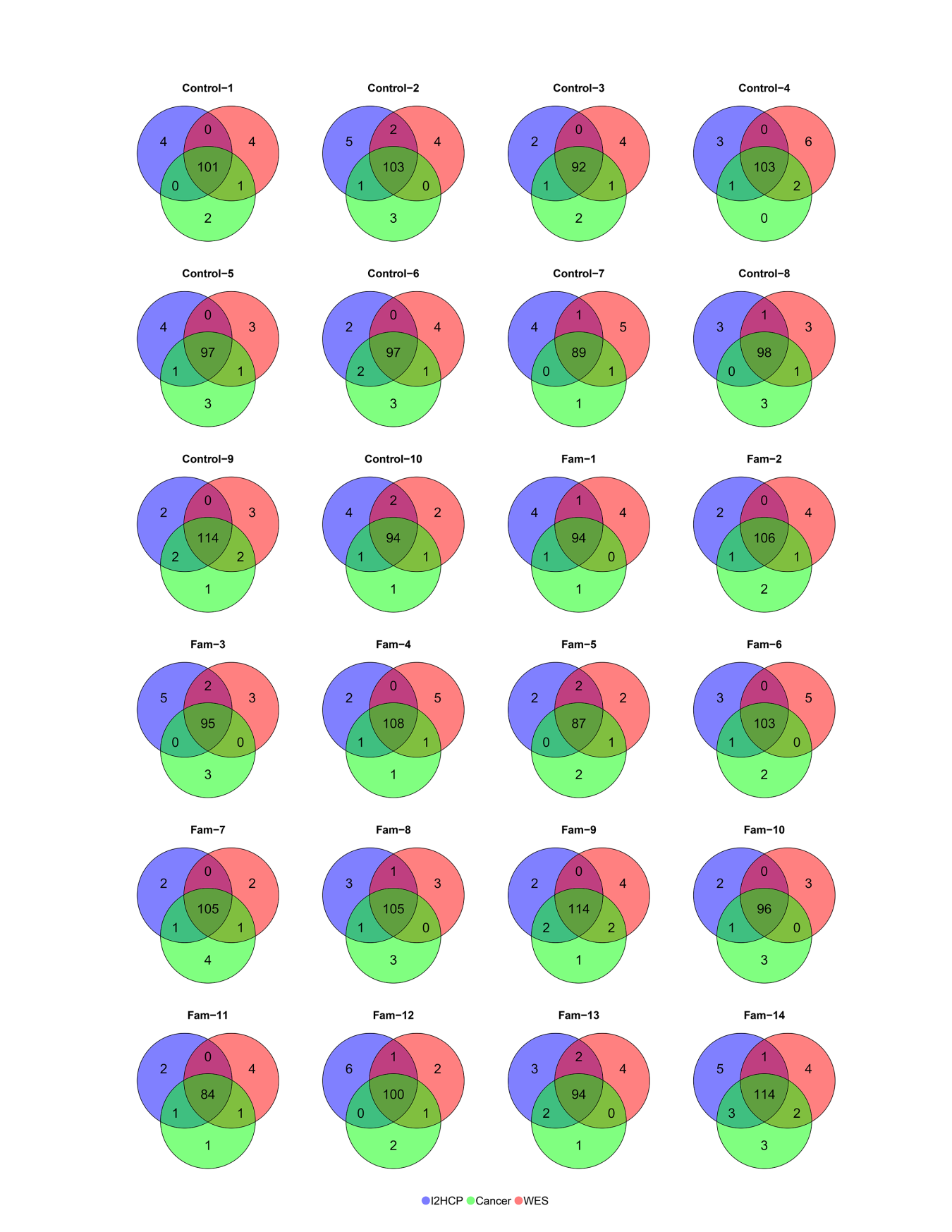


**Figure S6A. Schematic representation of the aberrant splicing caused by the *ATM* c.4776+2_4776+13delTAATAAAAATTT variant, which shows the abolition of the wild-type donor site of exon 31.** The cDNA amplification showed a double band, one corresponding to the wild-type transcript (400 bp) and the other one corresponding to a transcript lacking exon 31 (237 bp). Sequencing of the aberrant cDNA showed skipping of exon 31. WT, wild-type; Mut, mutant; P, puromycin-treated; NP; puromycin non-treated.

**
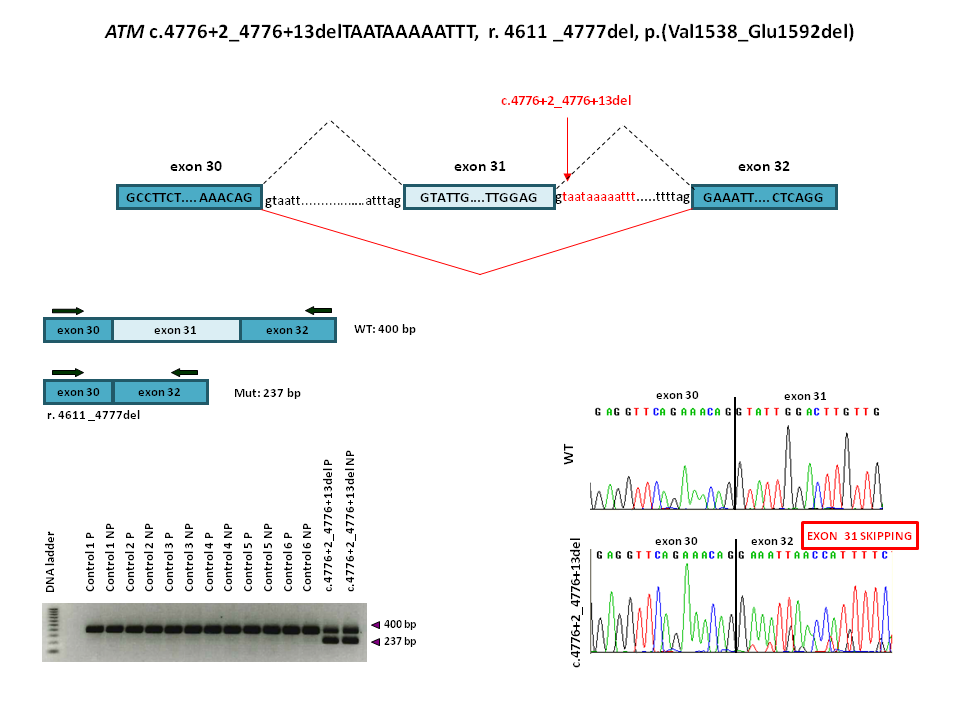
**

**Figure S6B. Schematic representation of the aberrant splicing caused by the *CHEK2* c.792+2T>C variant, which shows the abolition of the wild-type donor site of exon 6.** The RT-PCR analysis showed multiple bands (the 469-bp band corresponds to the predominant transcript) in the control samples. The 588-bp band belongs to an aberrant transcript showing a 119-bp insertion from intron 6. Sequencing of the aberrant cDNA showed the partial 119-bp insertion from intron 6. WT, wild-type; Mut, mutant; P, puromycin-treated; NP; puromycin non-treated.

**
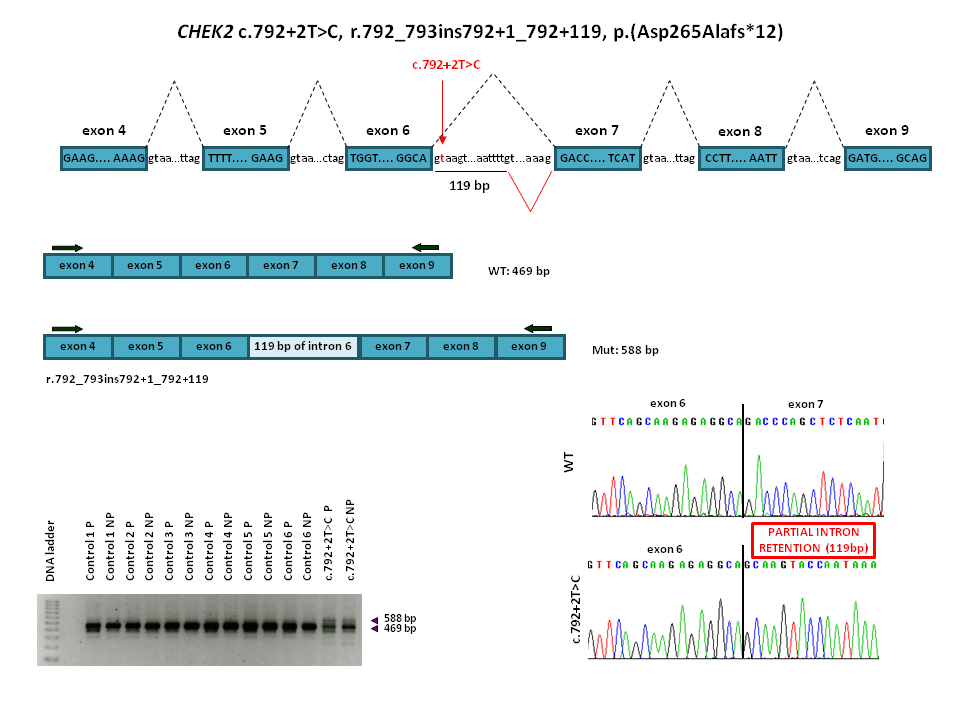
**

**Table S1. List of genes included in both panels.**

|  | **HUGO** | **MIM** | **Ensembl** | **HUGO** | **MIM** | **Ensembl** | **HUGO** | **MIM** | **Ensembl** |
| --- | --- | --- | --- | --- | --- | --- | --- | --- | --- |
| Common genes | *AIP* | 605555 | ENSG00000110711 | *FANCB* | 300515 | ENSG00000181544 | *PMS2* | 600259 | ENSG00000122512 |
|  | *ALK* | 105590 | ENSG00000171094 | *FANCC* | 613899 | ENSG00000158169 | *PRKAR1A* | 188830 | ENSG00000108946 |
|  | *APC* | 611731 | ENSG00000134982 | *FANCD2* | 613984 | ENSG00000144554 | *PTCH1* | 601309 | ENSG00000185920 |
|  | *ATM* | 607585 | ENSG00000149311 | *FANCE* | 613976 | ENSG00000112039 | *PTEN* | 601728 | ENSG00000171862 |
|  | *BAP1* | 603089 | ENSG00000163930 | *FANCF* | 613897 | ENSG00000183161 | *RAD51C* | 602774 | ENSG00000108384 |
|  | *BLM* | 604610 | ENSG00000197299 | *FANCG* | 602956 | ENSG00000221829 | *RAD51D* | 602954 | ENSG00000185379 |
|  | *BMPR1A* | 601299 | ENSG00000107779 | *FANCI* | 611360 | ENSG00000140525 | *RB1* | 614041 | ENSG00000139687 |
|  | *BRCA1* | 113705 | ENSG00000012048 | *FANCL* | 608111 | ENSG00000115392 | *RET* | 164761 | ENSG00000165731 |
|  | *BRCA2* | 600185 | ENSG00000139618 | *FANCM* | 609644 | ENSG00000187790 | *SBDS* | 607444 | ENSG00000126524 |
|  | *BRIP1* | 605882 | ENSG00000136492 | *FH* | 136850 | ENSG00000091483 | *SDHAF2* | 613019 | ENSG00000167985 |
|  | *BUB1B* | 602860 | ENSG00000156970 | *FLCN* | 607273 | ENSG00000154803 | *SDHB* | 185470 | ENSG00000117118 |
|  | *CDC73* | 607393 | ENSG00000134371 | *GPC3* | 300037 | ENSG00000147257 | *SDHC* | 602413 | ENSG00000143252 |
|  | *CDH1* | 192090 | ENSG00000039068 | *HNF1A* | 142410 | ENSG00000135100 | *SDHD* | 602690 | ENSG00000204370 |
|  | *CDK4* | 123829 | ENSG00000135446 | *HRAS* | 190020 | ENSG00000174775 | *SLX4* | 613278 | ENSG00000188827 |
|  | *CDKN1C* | 600856 | ENSG00000129757 | *KIT* | 164920 | ENSG00000157404 | *SMAD4* | 600993 | ENSG00000141646 |
|  | *CDKN2A* | 600160 | ENSG00000147889 | *MAX* | 154950 | ENSG00000125952 | *SMARCB1* | 601607 | ENSG00000099956 |
|  | *CHEK2* | 604373 | ENSG00000183765 | *MEN1* | 613733 | ENSG00000133895 | *STK11* | 602216 | ENSG00000118046 |
|  | *CYLD* | 605018 | ENSG00000083799 | *MET* | 164860 | ENSG00000105976 | *SUFU* | 607035 | ENSG00000107882 |
|  | *DDB2* | 600811 | ENSG00000134574 | *MLH1* | 120436 | ENSG00000076242 | *TMEM127* | 613403 | ENSG00000135956 |
|  | *DICER1* | 606241 | ENSG00000100697 | *MSH2* | 609309 | ENSG00000095002 | *TP53* | 191170 | ENSG00000141510 |
|  | *EPCAM* | 185535 | ENSG00000119888 | *MSH6* | 600678 | ENSG00000116062 | *TSC1* | 605284 | ENSG00000165699 |
|  | *ERCC2* | 126340 | ENSG00000104884 | *MUTYH* | 604933 | ENSG00000132781 | *TSC2* | 191092 | ENSG00000103197 |
|  | *ERCC3* | 133510 | ENSG00000163161 | *NBN* | 602667 | ENSG00000104320 | *VHL* | 608537 | ENSG00000134086 |
|  | *ERCC4* | 133520 | ENSG00000175595 | *NF1* | 162200 | ENSG00000196712 | *WRN* | 604611 | ENSG00000165392 |
|  | *ERCC5* | 133530 | ENSG00000134899 | *NF2* | 607379 | ENSG00000186575 | *WT1* | 607102 | ENSG00000184937 |
|  | *EXT1* | 608177 | ENSG00000182197 | *PALB2* | 610355 | ENSG00000083093 | *XPA* | 611153 | ENSG00000136936 |
|  | *EXT2* | 608210 | ENSG00000151348 | *PHOX2B* | 603851 | ENSG00000109132 | *XPC* | 613208 | ENSG00000154767 |
|  | *FANCA* | 607139 | ENSG00000187741 | *PMS1* | 600258 | ENSG00000064933 |  |  |  |
| Only in I2HCP | *ARAF* | 311010 | ENSG00000078061 | *MAP2K2* | 601263 | ENSG00000126934 | *PTPN11* | 176876 | ENSG00000179295 |
|  | *BARD1* | 601593 | ENSG00000138376 | *MLH3* | 604395 | ENSG00000119684 | *RAD50* | 604040 | ENSG00000113522 |
|  | *BRAF* | 164757 | ENSG00000157764 | *MN1* | 156100 | ENSG00000169184 | *RAD51* | 179617 | ENSG00000051180 |
|  | *CBL* | 165360 | ENSG00000110395 | *MRE11A* | 600814 | ENSG00000020922 | *RAF1* | 164760 | ENSG00000132155 |
|  | *CDKN2C* | 603369 | ENSG00000123080 | *MSH3* | 600887 | ENSG00000113318 | *RASA1* | 139150 | ENSG00000145715 |
|  | *DDB1* | 600045 | ENSG00000167986 | *NRAS* | 164790 | ENSG00000213281 | *RNASEL* | 180435 | ENSG00000135828 |
|  | *ELAC2* | 605367 | ENSG00000006744 | *PDGFB* | 190040 | ENSG00000100311 | *SHOC2* | 602775 | ENSG00000108061 |
|  | *ERCC6* | 609413 | ENSG00000225830 | *PDGFRA* | 173490 | ENSG00000134853 | *SMARCE1* | 603111 | ENSG00000073584 |
|  | *ERCC8* | 609412 | ENSG00000049167 | *POLD1* ^a^ | 174761 | ENSG00000062822 | *SOS1* | 182530 | ENSG00000115904 |
|  | *EXO1* | 606063 | ENSG00000174371 | *POLE* ^a^ | 174762 | ENSG00000177084 | *SPRED1* | 609291 | ENSG00000166068 |
|  | *KLLN* | 612105 | ENSG00000227268 | *POLH* | 603968 | ENSG00000170734 | *TGFBR2* | 190182 | ENSG00000163513 |
|  | *KRAS* | 190070 | ENSG00000133703 | *PPM1D* | 605100 | ENSG00000170836 | *TSHR* | 603372 | ENSG00000165409 |
|  | *MAP2K1* | 176872 | ENSG00000169032 | *PRSS1* | 276000 | ENSG00000204983 | *XRCC2* | 600375 | ENSG00000196584 |
| Only in TSCP | *CEBPA* | 116897 | ENSG00000245848 | *EZH2* | 601573 | ENSG00000106462 | *RECQL4* | 603780 | ENSG00000160957 |
|  | *CEP57* | 607951 | ENSG00000166037 | *GATA2* | 137295 | ENSG00000179348 | *RHBDF2* | 614404 | ENSG00000129667 |
|  | *DIS3L2* | 614184 | ENSG00000144535 | *NSD1* | 606681 | ENSG00000165671 | *RUNX1* | 151385 | ENSG00000159216 |
|  | *EGFR* | 131550 | ENSG00000146648 | *PRF1* | 170280 | ENSG00000180644 |  |  |  |

*^a^* Only the catalytic region is targeted

**Table S2. Main quality control (QC) data.** Results expressed as mean ± standard deviation.

| **QC summary** | | **I2HCP** | **TSCP** | **WES** |
| --- | --- | --- | --- | --- |
| Yield per sample | Passing filter reads | 7,326,270 ± 773,124 | 7,745,357 ± 1,973,522 | 67,234,215 ± 5,343,254 |
|  | Read lengths | 2 x 101 | 2 x 101 | 2 x 101 |
|  | Yield PF total bases | 1,479,906,599 ± 156,171,086 | 1,564,562,055 ± 398,651,381 | 13,581,311,472 ± 1,079,337,361 |
| Mapping | Unique-mapping reads | 90.28% ± 0.69 | 87.77% ± 2.33 | 94.77% ± 0.61 |
|  | Multiple-mapping reads | 7.13% ± 0.39 | 7.71% ± 0.40 | 3.36% ± 0.08 |
|  | Unmapped reads | 2.60% ± 0.40 | 4.52% ± 2.36 | 1.87% ± 0.64 |
| Pair statistics based on unique-mapping reads | Read pairs on different strands | 99.25% ± 0.147 | 99.81% ± 0.013 | 99.77% ± 0.058 |
|  | Read pairs on same strand | 0.03% ± 0.005 | 0.01% ± 0.001 | 0.01% ± 0.002 |
|  | Read pairs on different contigs | 0.73% ± 0.143 | 0.18% ± 0.012 | 0.22% ± 0.057 |
| Duplicates | Duplicate percentage | 2.76% ± 0.84 | 66.16% ± 4.70 | 7.33% ± 4.23 |

**Table S3. Comparison of the Diagnostic Region Of Interest (DxROI^a^) and the target regions supplied for each approach.**

| Bases of the DxROIs covered by the target region of each approach | | | | | | |
| --- | --- | --- | --- | --- | --- | --- |
|  | I2HCP | | TSCP | | WES | |
| 83-gene DxROI unique bases: 272,797 | 271,850 | 99.65% | 219,639 | 80.51% | 237,702 | 87.14% |
| 132-gene DxROI unique bases: 415,175 | 370,655 | 89.28% | 246,674 | 59.41% | 361,269 | 87.02% |
| Intersections of all three target regions and percentages versus the approach in the column | | | | | | |
|  | I2HCP | | TSCP | | WES | |
| I2HCP | 396,958 | 100.00% | 219,993 | 87.81% | 336,559 | 0.67% |
| TSCP | 219,993 | 55.42% | 250,524 | 100.00% | 230,417 | 0.46% |
| WES | 336,559 | 84.78% | 230,417 | 91.97% | 50,390,601 | 100.00% |
| Intersections of the bases of the DxROIs covered by each target region with the remaining target regions | | | | | | |
| 83-gene DxROI | I2HCP | | TSCP | | WES | |
| I2HCP | 271,850 | 100.00% | 219,420 | 99.90% | 237,094 | 99.74% |
| TSCP | 219,420 | 80.71% | 219,639 | 100.00% | 201,573 | 84.80% |
| WES | 237,094 | 87.22% | 201,573 | 91.77% | 237,702 | 100.00% |
| 132-gene DxROI | I2HCP | | TSCP | | WES | |
| I2HCP | 370,655 | 100.00% | 219,979 | 89.18% | 322,210 | 89.19% |
| TSCP | 219,979 | 59.35% | 246,674 | 100.00% | 226,822 | 62.78% |
| WES | 322,210 | 86.93% | 226,822 | 91.95% | 361,269 | 100.00% |

*^a^* We define the Diagnostic Region Of Interest (DxROI) as the sum of all CCDS (consensus coding sequence) bases of the selected genes plus 20 bp (either intronic, 3’UTR or 5’UTR) surrounding each coding fragment. As we have defined a common gene set (83 CCDS genes covered by both panels and WES) and a comprehensive gene set (132 CCDS genes covered by any of the panels, and the WES), we analyze these two DxROIs.

**Table S4. Theoretical and observed coverage detailed gene by gene.** Target region coverage of the 132-gene DxROI, mean depth and percentage of C30 bases for each panel gene (median from the 24 samples).

| **Gene** | **DxROI Size** | **DxROI theoretical coverage by gene** | | | | | | **DxROI observed coverage by gene** | | | | | |
| --- | --- | --- | --- | --- | --- | --- | --- | --- | --- | --- | --- | --- | --- |
|  |  | **I2HCP** | | **TSCP** | | **WES** | | **I2HCP** | | **TSCP** | | **WES** | |
|  |  | **Target reg*io*n in the DxROI** | **%** | **Target region in the DxROI** | **%** | **Target region in the DxROI** | **%** | **Mean read depth** | **C30 base %** | **Mean read depth** | **C30 base %** | **Mean read depth** | **C30 base %** |
| *AIP* | 1233 | 1233 | 100.00 | 1033 | 83.78 | 1046 | 84.83 | 374.34 | 100.00 | 579.35 | 100.00 | 103.06 | 96.55 |
| *ALK* | 6023 | 5994 | 99.52 | 4892 | 81.22 | 5298 | 87.96 | 484.05 | 100.00 | 453.52 | 100.00 | 120.62 | 96.20 |
| *APC* | 9132 | 9132 | 100.00 | 8547 | 93.59 | 8780 | 96.15 | 632.15 | 100.00 | 437.00 | 100.00 | 208.86 | 96.80 |
| *ARAF ^a^* | 2425 | 2425 | 100.00 | 0 | 0.00 | 2163 | 89.20 | 446.40 | 100.00 | 0.00 | 0.00 | 135.64 | 100.00 |
| *ATM* | 11651 | 11651 | 100.00 | 9233 | 79.25 | 10034 | 86.12 | 497.05 | 100.00 | 368.86 | 100.00 | 114.31 | 94.45 |
| *BAP1* | 2870 | 2853 | 99.41 | 2207 | 76.90 | 2494 | 86.90 | 444.01 | 100.00 | 392.99 | 100.00 | 127.59 | 97.55 |
| *BARD1 ^a^* | 2774 | 2763 | 99.60 | 0 | 0.00 | 2664 | 96.03 | 593.54 | 100.00 | 0.00 | 0.00 | 175.28 | 99.40 |
| *BLM* | 5094 | 5094 | 100.00 | 4275 | 83.92 | 4404 | 86.45 | 525.83 | 100.00 | 384.65 | 100.00 | 140.40 | 98.80 |
| *BMPR1A* | 2039 | 2039 | 100.00 | 1610 | 78.96 | 950 | 46.59 | 553.77 | 100.00 | 349.26 | 100.00 | 119.38 | 96.50 |
| *BRAF ^a^* | 3021 | 3003 | 99.40 | 0 | 0.00 | 2119 | 70.14 | 420.38 | 95.85 | 0.00 | 0.00 | 74.64 | 72.85 |
| *BRCA1* | 6578 | 6555 | 99.65 | 5614 | 85.35 | 6193 | 94.15 | 673.08 | 100.00 | 396.33 | 98.40 | 229.20 | 96.20 |
| *BRCA2* | 11297 | 11297 | 100.00 | 10283 | 91.02 | 10656 | 94.33 | 609.70 | 100.00 | 363.37 | 100.00 | 153.85 | 97.35 |
| *BRIP1* | 4510 | 4491 | 99.58 | 3769 | 83.57 | 3975 | 88.14 | 573.15 | 100.00 | 335.13 | 100.00 | 152.15 | 96.85 |
| *BUB1B* | 4073 | 4073 | 100.00 | 3176 | 77.98 | 3615 | 88.76 | 553.52 | 100.00 | 381.80 | 100.00 | 143.28 | 98.90 |
| *CBL ^a^* | 3361 | 3361 | 100.00 | 0 | 0.00 | 3043 | 90.54 | 571.63 | 100.00 | 0.00 | 0.00 | 128.81 | 93.05 |
| *CDC73* | 2276 | 2276 | 100.00 | 1613 | 70.87 | 2066 | 90.77 | 471.80 | 100.00 | 337.20 | 100.00 | 90.52 | 94.70 |
| *CDH1* | 3289 | 3289 | 100.00 | 2665 | 81.03 | 2924 | 88.90 | 569.68 | 97.30 | 432.86 | 100.00 | 91.01 | 96.40 |
| *CDK4* | 1192 | 1185 | 99.41 | 919 | 77.10 | 1087 | 91.19 | 605.48 | 100.00 | 470.00 | 100.00 | 129.39 | 100.00 |
| *CDKN1C* | 1031 | 1029 | 99.81 | 953 | 92.43 | 793 | 76.92 | 161.78 | 83.80 | 153.44 | 84.75 | 57.97 | 75.00 |
| *CDKN2A* | 911 | 906 | 99.45 | 668 | 73.33 | 756 | 82.99 | 345.84 | 100.00 | 321.32 | 90.50 | 103.16 | 98.65 |
| *CDKN2C ^a^* | 587 | 587 | 100.00 | 0 | 0.00 | 496 | 84.50 | 723.83 | 100.00 | 0.00 | 0.00 | 138.60 | 100.00 |
| *CEBPA ^b^* | 1117 | 0 | 0.00 | 1078 | 96.51 | 841 | 75.29 | 0.05 | 0.00 | 115.85 | 69.40 | 148.16 | 84.70 |
| *CEP57 ^b^* | 2001 | 0 | 0.00 | 1514 | 75.66 | 1756 | 87.76 | 0.05 | 0.00 | 311.14 | 97.10 | 106.69 | 85.95 |
| *CHEK2* | 2361 | 2346 | 99.36 | 1646 | 69.72 | 1768 | 74.88 | 460.53 | 100.00 | 343.43 | 100.00 | 81.53 | 73.25 |
| *CYLD* | 3551 | 3551 | 100.00 | 2888 | 81.33 | 3257 | 91.72 | 571.19 | 100.00 | 366.57 | 100.00 | 125.10 | 89.90 |
| *DDB1 ^a^* | 4503 | 4476 | 99.40 | 0 | 0.00 | 3901 | 86.63 | 601.35 | 100.00 | 422.29 | 100.00 | 119.43 | 94.45 |
| *DDB2* | 1684 | 1684 | 100.00 | 1294 | 76.84 | 1418 | 84.20 | 578.87 | 100.00 | 420.11 | 100.00 | 147.55 | 98.05 |
| *DICER1* | 6809 | 6783 | 99.62 | 5795 | 85.11 | 5906 | 86.74 | 563.35 | 100.00 | 438.53 | 100.00 | 172.22 | 99.65 |
| *DIS3L2 ^b^* | 3777 | 0 | 0.00 | 2678 | 70.90 | 3350 | 88.69 | 0.10 | 0.00 | 278.95 | 92.80 | 131.67 | 95.15 |
| *EGFR ^b^* | 5089 | 0 | 0.00 | 3661 | 71.94 | 4381 | 86.09 | 10.64 | 4.40 | 364.95 | 93.60 | 132.42 | 100.00 |
| *ELAC2 ^a^* | 3441 | 3417 | 99.30 | 0 | 0.00 | 3088 | 89.74 | 505.33 | 100.00 | 0.00 | 0.00 | 113.41 | 95.40 |
| *EPCAM* | 1305 | 1305 | 100.00 | 954 | 73.10 | 1099 | 84.21 | 467.18 | 100.00 | 296.46 | 100.00 | 76.29 | 92.60 |
| *ERCC2* | 3296 | 3272 | 99.27 | 2306 | 69.96 | 2981 | 90.44 | 490.47 | 100.00 | 472.65 | 97.20 | 118.17 | 97.25 |
| *ERCC3* | 2949 | 2934 | 99.49 | 2364 | 80.16 | 2524 | 85.59 | 554.53 | 100.00 | 445.32 | 100.00 | 99.69 | 92.25 |
| *ERCC4* | 3191 | 3191 | 100.00 | 2762 | 86.56 | 2837 | 88.91 | 568.06 | 100.00 | 407.93 | 100.00 | 150.50 | 99.45 |
| *ERCC5* | 4161 | 4161 | 100.00 | 3576 | 85.94 | 3753 | 90.19 | 559.05 | 100.00 | 403.19 | 100.00 | 165.21 | 99.90 |
| *ERCC6 ^a^* | 5282 | 5262 | 99.62 | 0 | 0.00 | 4894 | 92.65 | 631.77 | 100.00 | 0.00 | 0.00 | 198.57 | 99.80 |
| *ERCC8 ^a^* | 1671 | 1659 | 99.28 | 0 | 0.00 | 1527 | 91.38 | 433.10 | 100.00 | 0.00 | 0.00 | 71.06 | 72.65 |
| *EXO1 ^a^* | 3061 | 3061 | 100.00 | 0 | 0.00 | 2774 | 90.62 | 581.10 | 100.00 | 0.00 | 0.00 | 160.72 | 100.00 |
| *EXT1* | 2681 | 2670 | 99.59 | 2252 | 84.00 | 2416 | 90.12 | 536.63 | 100.00 | 427.35 | 100.00 | 91.02 | 94.10 |
| *EXT2* | 2949 | 2949 | 100.00 | 2270 | 76.98 | 2596 | 88.03 | 573.09 | 100.00 | 404.40 | 95.50 | 144.09 | 98.85 |
| *EZH2 ^b^* | 3016 | 0 | 0.00 | 2275 | 75.43 | 2586 | 85.74 | 0.09 | 0.00 | 265.37 | 100.00 | 142.06 | 97.80 |
| *FANCA* | 6133 | 6089 | 99.28 | 4411 | 71.92 | 5415 | 88.29 | 506.06 | 100.00 | 430.85 | 100.00 | 101.66 | 96.40 |
| *FANCB* | 2900 | 2892 | 99.72 | 2588 | 89.24 | 2695 | 92.93 | 457.34 | 100.00 | 408.68 | 100.00 | 112.72 | 95.45 |
| *FANCC* | 2237 | 2223 | 99.37 | 1691 | 75.59 | 2045 | 91.42 | 519.29 | 100.00 | 392.12 | 100.00 | 109.17 | 99.50 |
| *FANCD2* | 6211 | 6211 | 100.00 | 4458 | 71.78 | 5058 | 81.44 | 498.42 | 100.00 | 331.22 | 100.00 | 109.89 | 90.30 |
| *FANCE* | 2011 | 2011 | 100.00 | 1621 | 80.61 | 1766 | 87.82 | 426.00 | 85.70 | 424.90 | 100.00 | 114.07 | 85.70 |
| *FANCF* | 1165 | 1164 | 99.91 | 1126 | 96.65 | 1129 | 96.91 | 538.85 | 100.00 | 529.25 | 100.00 | 223.80 | 100.00 |
| *FANCG* | 2429 | 2415 | 99.42 | 1883 | 77.52 | 2112 | 86.95 | 558.86 | 100.00 | 565.43 | 100.00 | 125.32 | 100.00 |
| *FANCI* | 5467 | 5467 | 100.00 | 4024 | 73.61 | 4961 | 90.74 | 625.92 | 100.00 | 383.09 | 100.00 | 150.20 | 98.45 |
| *FANCL* | 1703 | 1689 | 99.18 | 1157 | 67.94 | 1550 | 91.02 | 426.93 | 100.00 | 388.48 | 100.00 | 80.03 | 96.25 |
| *FANCM* | 7067 | 7067 | 100.00 | 6170 | 87.31 | 6295 | 89.08 | 568.24 | 100.00 | 367.76 | 100.00 | 118.46 | 91.30 |
| *FH* | 1933 | 1923 | 99.48 | 1543 | 79.82 | 1370 | 70.87 | 438.55 | 100.00 | 381.50 | 100.00 | 132.24 | 85.90 |
| *FLCN* | 2338 | 2327 | 99.53 | 1751 | 74.89 | 2043 | 87.38 | 541.34 | 100.00 | 530.56 | 100.00 | 125.33 | 100.00 |
| *GATA2 ^b^* | 1643 | 0 | 0.00 | 1448 | 88.13 | 1457 | 88.68 | 0.11 | 0.00 | 206.61 | 100.00 | 109.89 | 97.60 |
| *GPC3* | 2172 | 2163 | 99.59 | 1751 | 80.62 | 1924 | 88.58 | 452.97 | 100.00 | 435.97 | 100.00 | 119.64 | 96.05 |
| *HNF1A* | 2296 | 2296 | 100.00 | 1906 | 83.01 | 1934 | 84.23 | 480.37 | 100.00 | 535.80 | 100.00 | 135.84 | 99.00 |
| *HRAS* | 833 | 828 | 99.40 | 574 | 68.91 | 792 | 95.08 | 535.17 | 100.00 | 523.52 | 100.00 | 121.92 | 100.00 |
| *KIT* | 3771 | 3771 | 100.00 | 2952 | 78.28 | 3317 | 87.96 | 590.69 | 100.00 | 396.51 | 100.00 | 147.40 | 100.00 |
| *KLLN ^a^* | 577 | 576 | 99.83 | 0 | 0.00 | 519 | 89.95 | 409.91 | 100.00 | 0.00 | 0.00 | 179.19 | 100.00 |
| *KRAS ^a^* | 887 | 882 | 99.44 | 0 | 0.00 | 778 | 87.71 | 504.83 | 100.00 | 0.00 | 0.00 | 60.28 | 100.00 |
| *MAP2K1 ^a^* | 1622 | 1622 | 100.00 | 0 | 0.00 | 1083 | 66.77 | 571.48 | 100.00 | 0.00 | 0.00 | 99.40 | 83.50 |
| *MAP2K2 ^a^* | 1643 | 1632 | 99.33 | 0 | 0.00 | 1069 | 65.06 | 403.23 | 92.00 | 0.00 | 0.00 | 82.75 | 89.75 |
| *MAX* | 1021 | 958 | 93.83 | 488 | 47.80 | 945 | 92.56 | 430.80 | 100.00 | 205.04 | 66.90 | 83.78 | 94.40 |
| *MEN1* | 2208 | 2199 | 99.59 | 1857 | 84.10 | 1816 | 82.25 | 505.06 | 100.00 | 527.48 | 100.00 | 103.24 | 95.30 |
| *MET* | 5027 | 4987 | 99.20 | 4247 | 84.48 | 4621 | 91.92 | 633.55 | 100.00 | 306.28 | 100.00 | 183.12 | 98.85 |
| *MLH1* | 3031 | 3031 | 100.00 | 2290 | 75.55 | 2739 | 90.37 | 540.82 | 99.70 | 371.12 | 100.00 | 163.66 | 99.30 |
| *MLH3 ^a^* | 4842 | 4830 | 99.75 | 0 | 0.00 | 4647 | 95.97 | 657.67 | 100.00 | 0.00 | 0.00 | 209.83 | 100.00 |
| *MN1 ^a^* | 4043 | 4041 | 99.95 | 0 | 0.00 | 3676 | 90.92 | 204.01 | 99.45 | 0.05 | 0.00 | 141.64 | 98.20 |
| *MRE11A ^a^* | 2887 | 2868 | 99.34 | 0 | 0.00 | 2345 | 81.23 | 530.18 | 100.00 | 0.00 | 0.00 | 58.82 | 83.90 |
| *MSH2* | 3445 | 3445 | 100.00 | 2821 | 81.89 | 2928 | 84.99 | 539.87 | 100.00 | 350.65 | 100.00 | 92.96 | 91.75 |
| *MSH3 ^a^* | 4374 | 4374 | 100.00 | 0 | 0.00 | 3763 | 86.03 | 475.25 | 100.00 | 0.01 | 0.00 | 124.08 | 95.05 |
| *MSH6* | 4483 | 4483 | 100.00 | 4093 | 91.30 | 4322 | 96.41 | 625.54 | 100.00 | 452.64 | 100.00 | 202.89 | 99.05 |
| *MUTYH* | 2281 | 2265 | 99.30 | 1666 | 73.04 | 2130 | 93.38 | 628.05 | 100.00 | 604.00 | 100.00 | 149.51 | 100.00 |
| *NBN* | 2905 | 2889 | 99.45 | 2281 | 78.52 | 2580 | 88.81 | 512.40 | 100.00 | 328.19 | 100.00 | 88.80 | 93.50 |
| *NF1* | 10901 | 10901 | 100.00 | 8578 | 78.69 | 8187 | 75.10 | 552.97 | 99.10 | 350.84 | 100.00 | 119.23 | 87.35 |
| *NF2* | 2504 | 2504 | 100.00 | 1804 | 72.04 | 2239 | 89.42 | 577.55 | 100.00 | 321.86 | 100.00 | 92.01 | 98.00 |
| *NRAS ^a^* | 730 | 726 | 99.45 | 0 | 0.00 | 687 | 94.11 | 551.29 | 100.00 | 0.00 | 0.00 | 168.86 | 100.00 |
| *NSD1 ^b^* | 8971 | 0 | 0.00 | 8113 | 90.44 | 8459 | 94.29 | 0.12 | 0.00 | 411.29 | 100.00 | 176.98 | 100.00 |
| *PALB2* | 4081 | 4068 | 99.68 | 3574 | 87.58 | 3800 | 93.11 | 596.49 | 100.00 | 399.78 | 100.00 | 190.57 | 99.90 |
| *PDGFB ^a^* | 1024 | 1017 | 99.32 | 0 | 0.00 | 849 | 82.91 | 288.65 | 100.00 | 0.00 | 0.00 | 91.99 | 99.45 |
| *PDGFRA ^a^* | 4150 | 4150 | 100.00 | 0 | 0.00 | 3619 | 87.20 | 629.14 | 100.00 | 0.00 | 0.00 | 138.02 | 100.00 |
| *PHOX2B* | 1065 | 1062 | 99.72 | 948 | 89.01 | 805 | 75.59 | 353.44 | 100.00 | 241.41 | 89.75 | 80.57 | 99.90 |
| *PMS1* | 3279 | 3279 | 100.00 | 2811 | 85.73 | 2872 | 87.59 | 526.05 | 100.00 | 354.59 | 100.00 | 94.09 | 91.45 |
| *PMS2* | 3189 | 3174 | 99.53 | 2604 | 81.66 | 1999 | 62.68 | 345.19 | 99.00 | 281.80 | 96.30 | 68.32 | 55.50 |
| *POLD1* *^a^* | 4364 | 1417 | 32.47 | 0 | 0.00 | 3785 | 86.73 | 132.38 | 39.20 | 0.00 | 0.00 | 98.10 | 83.95 |
| *POLE ^a^* | 8821 | 1459 | 16.54 | 0 | 0.00 | 7620 | 86.38 | 92.97 | 16.70 | 0.01 | 0.00 | 109.55 | 97.75 |
| *POLH ^a^* | 2542 | 2542 | 100.00 | 0 | 0.00 | 2426 | 95.44 | 587.22 | 100.00 | 0.00 | 0.00 | 159.95 | 97.65 |
| *PPM1D ^a^* | 2058 | 2058 | 100.00 | 559 | 27.16 | 1941 | 94.31 | 576.88 | 100.00 | 218.81 | 88.60 | 186.22 | 99.55 |
| *PRF1 ^b^* | 1748 | 0 | 0.00 | 1670 | 95.54 | 1636 | 93.59 | 0.14 | 0.00 | 632.60 | 100.00 | 139.24 | 98.85 |
| *PRKAR1A* | 1627 | 1546 | 95.02 | 1156 | 71.05 | 1485 | 91.27 | 504.98 | 95.00 | 315.25 | 95.00 | 85.91 | 85.50 |
| *PRSS1 ^a^* | 944 | 944 | 100.00 | 0 | 0.00 | 553 | 58.58 | 482.96 | 100.00 | 0.00 | 0.00 | 168.62 | 100.00 |
| *PTCH1* | 5545 | 5283 | 95.28 | 4364 | 78.70 | 4603 | 83.01 | 519.66 | 100.00 | 394.10 | 95.70 | 124.77 | 98.05 |
| *PTEN* | 1572 | 1572 | 100.00 | 1221 | 77.67 | 1227 | 78.05 | 478.00 | 100.00 | 310.97 | 100.00 | 122.83 | 99.15 |
| *PTPN11 ^a^* | 2386 | 2386 | 100.00 | 0 | 0.00 | 951 | 39.86 | 482.65 | 97.70 | 0.00 | 0.00 | 104.95 | 85.65 |
| *RAD50 ^a^* | 4939 | 4416 | 89.41 | 0 | 0.00 | 4028 | 81.55 | 488.09 | 100.00 | 0.00 | 0.00 | 104.90 | 90.00 |
| *RAD51 ^a^* | 1541 | 1541 | 100.00 | 0 | 0.00 | 1313 | 85.20 | 520.60 | 100.00 | 309.82 | 86.40 | 111.41 | 93.40 |
| *RAD51C* | 1495 | 1495 | 100.00 | 1140 | 76.25 | 1331 | 89.03 | 521.19 | 100.00 | 353.99 | 100.00 | 126.98 | 93.40 |
| *RAD51D* | 1606 | 1595 | 99.32 | 997 | 62.08 | 1274 | 79.33 | 511.46 | 100.00 | 366.15 | 86.40 | 104.36 | 100.00 |
| *RAF1 ^a^* | 2587 | 2571 | 99.38 | 0 | 0.00 | 2206 | 85.27 | 581.38 | 100.00 | 0.03 | 0.00 | 120.97 | 100.00 |
| *RASA1 ^a^* | 4192 | 4192 | 100.00 | 0 | 0.00 | 3813 | 90.96 | 455.66 | 100.00 | 0.00 | 0.00 | 91.98 | 85.40 |
| *RB1* | 3867 | 3867 | 100.00 | 2814 | 72.77 | 3395 | 87.79 | 406.39 | 100.00 | 299.47 | 100.00 | 77.14 | 75.20 |
| *RET* | 4177 | 4177 | 100.00 | 3365 | 80.56 | 3595 | 86.07 | 466.39 | 100.00 | 444.56 | 100.00 | 118.02 | 92.70 |
| *RHBDF2 ^b^* | 3251 | 0 | 0.00 | 2588 | 79.61 | 2879 | 88.56 | 0.20 | 0.00 | 326.91 | 97.15 | 87.98 | 99.00 |
| *RNASEL ^a^* | 2466 | 2460 | 99.76 | 0 | 0.00 | 2255 | 91.44 | 664.37 | 100.00 | 0.00 | 0.00 | 171.01 | 98.55 |
| *RUNX1 ^b^* | 1848 | 0 | 0.00 | 1451 | 78.52 | 1699 | 91.94 | 0.17 | 0.00 | 178.78 | 88.45 | 103.28 | 95.75 |
| *SBDS* | 953 | 948 | 99.48 | 758 | 79.54 | 640 | 67.16 | 493.61 | 100.00 | 274.96 | 100.00 | 123.17 | 79.70 |
| *SDHAF2* | 661 | 661 | 100.00 | 505 | 76.40 | 497 | 75.19 | 521.89 | 100.00 | 388.44 | 100.00 | 158.52 | 100.00 |
| *SDHB* | 1163 | 1155 | 99.31 | 851 | 73.17 | 1070 | 92.00 | 472.58 | 100.00 | 289.46 | 100.00 | 112.12 | 100.00 |
| *SDHC* | 857 | 857 | 100.00 | 516 | 60.21 | 424 | 49.47 | 431.11 | 100.00 | 360.84 | 100.00 | 81.69 | 83.30 |
| *SDHD* | 798 | 798 | 100.00 | 484 | 60.65 | 225 | 28.20 | 438.35 | 100.00 | 261.79 | 80.20 | 41.56 | 49.40 |
| *SHOC2 ^a^* | 2069 | 2069 | 100.00 | 0 | 0.00 | 1790 | 86.52 | 572.06 | 100.00 | 0.00 | 0.00 | 159.94 | 100.00 |
| *SLX4* | 6065 | 6051 | 99.77 | 5519 | 91.00 | 5620 | 92.66 | 490.57 | 100.00 | 570.53 | 100.00 | 150.95 | 96.20 |
| *SMAD4* | 2099 | 2099 | 100.00 | 1670 | 79.56 | 1835 | 87.42 | 523.28 | 100.00 | 361.51 | 100.00 | 107.01 | 96.70 |
| *SMARCB1* | 1518 | 1518 | 100.00 | 1167 | 76.88 | 1287 | 84.78 | 530.24 | 100.00 | 391.04 | 100.00 | 146.94 | 100.00 |
| *SMARCE1 ^a^* | 1636 | 1626 | 99.39 | 0 | 0.00 | 771 | 47.13 | 596.36 | 100.00 | 0.00 | 0.00 | 116.88 | 83.80 |
| *SOS1 ^a^* | 4922 | 4899 | 99.53 | 0 | 0.00 | 4395 | 89.29 | 495.60 | 100.00 | 0.00 | 0.00 | 111.85 | 91.90 |
| *SPRED1 ^a^* | 1615 | 1615 | 100.00 | 0 | 0.00 | 1541 | 95.42 | 573.01 | 100.00 | 0.00 | 0.00 | 181.36 | 94.60 |
| *STK11* | 1662 | 1662 | 100.00 | 1311 | 78.88 | 1456 | 87.61 | 462.05 | 100.00 | 425.58 | 100.00 | 98.33 | 96.85 |
| *SUFU* | 1981 | 1981 | 100.00 | 1467 | 74.05 | 1709 | 86.27 | 507.38 | 100.00 | 371.17 | 97.70 | 112.35 | 99.20 |
| *TGFBR2* *^a^* | 2099 | 2099 | 100.00 | 0 | 0.00 | 1961 | 93.43 | 580.88 | 100.00 | 0.00 | 0.00 | 156.91 | 100.00 |
| *TMEM127* | 837 | 834 | 99.64 | 720 | 86.02 | 709 | 84.71 | 399.87 | 100.00 | 179.34 | 100.00 | 84.72 | 100.00 |
| *TP53* | 1743 | 1732 | 99.37 | 1192 | 68.39 | 1541 | 88.41 | 506.76 | 100.00 | 414.45 | 100.00 | 90.08 | 92.60 |
| *TSC1* | 4335 | 4314 | 99.52 | 3516 | 81.11 | 3828 | 88.30 | 576.19 | 100.00 | 420.42 | 100.00 | 124.92 | 94.40 |
| *TSC2* | 7064 | 7064 | 100.00 | 5465 | 77.36 | 6271 | 88.77 | 534.80 | 100.00 | 499.37 | 100.00 | 101.46 | 94.65 |
| *TSHR ^a^* | 2868 | 2819 | 98.29 | 0 | 0.00 | 2600 | 90.66 | 671.12 | 100.00 | 521.70 | 100.00 | 201.76 | 98.80 |
| *VHL* | 762 | 762 | 100.00 | 645 | 84.65 | 677 | 88.85 | 407.10 | 100.00 | 377.80 | 100.00 | 127.19 | 96.30 |
| *WRN* | 5659 | 5659 | 100.00 | 4333 | 76.57 | 5126 | 90.58 | 477.77 | 100.00 | 321.42 | 100.00 | 125.30 | 94.90 |
| *WT1* | 2004 | 1993 | 99.45 | 1564 | 78.04 | 1809 | 90.27 | 373.17 | 100.00 | 292.99 | 97.50 | 72.46 | 89.00 |
| *XPA* | 1062 | 1056 | 99.44 | 828 | 77.97 | 910 | 85.69 | 365.61 | 100.00 | 305.39 | 100.00 | 59.93 | 93.75 |
| *XPC* | 3463 | 3447 | 99.54 | 2839 | 81.98 | 3143 | 90.76 | 548.80 | 100.00 | 457.45 | 100.00 | 149.94 | 99.55 |
| *XRCC2 ^a^* | 963 | 960 | 99.69 | 0 | 0.00 | 870 | 90.34 | 634.43 | 100.00 | 0.00 | 0.00 | 211.42 | 87.30 |

*^a^* Not targeted by TSCP. *^b^* Not targeted by I2HCP.

**Table S5**. **Review of the putative pathogenic mutations identified in the discovery set.**

| **Gene** | **PPM** | **Putative RNA/protein effect** | **Pop. allele freq. (database)** | **Domain / region** | **Variant information literature** | **Variant information databases** |
| --- | --- | --- | --- | --- | --- | --- |
| *ATM* | c.4776+2_4776+13delTAATAAAAATTT | Exon31 skipping | 0.00083%  (ExAC) | Armadillo-type fold | - | (“dbSNP: rs762838462”) |
| *BARD1* | c.1921C>T | p.(Arg641*) | 0.00082%  (ExAC) | BRCT domain | Associated with triple-negative breast cancer patients^1^  Detected in a patient with neuroblastoma^2^ | Classified as pathogenic for hereditary cancer-predisposing syndrome (“ClinVAr: SCV000185168.2”; “dbSNP: rs587781948”) |
| *CHEK2* | c.792+2T>C | Exon 6 skipping | 0.00%  (ExAC) | Protein kinase-like | - | Classified as likely pathogenic for familial cancer of breast (“ClinVAR: RCV000205137.1”; “dbSNP: rs545982789”) |
| *ERCC3* | c.325C>T | p.(Arg109*) | Eur. Am.: 0.07% Afr. Am.: 0.00% (ESP)  0.05% (ExAC) | - | Found in *BRCA1/2* positive and negative patients from cancer genetics clinics^3^ | (“dbSNP: rs34295337”) |
| *FANCL* | c.1111_1114dupATTA | p.(Ile372Asnfs*13) | Eur. Am.: 0.35% Afr. Am.: 0.05% (ESP); 0.29%  (ExAC) | C-terminal | Associated with an increased risk of esophageal cancer^4^ | Classified as pathogenic for Fanconi anemia (“ClinVAr: SCV000247355.1”; “dbSNP: rs759217526”) |
| *FANCM* | c.5791C>T | r.[5791c>u, 5717_6008del] p.[Arg1931*, Gly1906Alafs*129]  Partial skipping of exon 22 | Eur. Am.: 0.09% Afr. Am.: 0.07% (Esp); 0.09%  (ExAC) | Restriction endonuclease type II-like, ERCC4 domain, DNA repair nuclease, XPF-type/Helicase | Induces exon skipping, affects DNA repair activity and is a familial breast cancer risk factor. Influences the DNA repair activity of the FANCM protein^5^ | (“dbSNP: rs144567652”) |
| *MSH2* | c.2785C>T | p.(Arg929*) | 0.01%  (ExAC) | - | Classified as an uknown significance variant according to IHC and family history information in Lynch syndrome families^6^ | Classified as pathogenic for Lynch syndrome (“InSIGHT: 1002161”; “ClinVAR: SCV000107577”) and classified as uknown significance variant for Lynch syndrome (“ClinVAR: SCV000166279”) |

Acronyms: PPM, putative pathogenic mutation; Pop. allele freq., population frequency; LOH; loss of heterozygosity; ESP, Exome Sequencing Project; ExAC, The Exome Aggregation Consortium; NI, no information available; IHC; immunohistochemistry

**Table S6. List of non-synonymous coding and splice-site variants with population frequency below 1% identified in the discovery set in the panel analysis.**

| **Family** | **Gene** | **MIM** | **Transcript** | **cDNA** | **Protein** |
| --- | --- | --- | --- | --- | --- |
| Fam-1 | *ATM* | 607585 | NM_000051.3 | c.4776+2_4776+13delTAATAAAAATTT *^a^* | p.? |
|  | *FANCM* | 609644 | NM_020937.2 | c.527C>T | p.(Thr176Ile) |
|  | *MLH1* | 120436 | NM_000249.3 | c.1231A>G | p.(Ile411Val) |
|  | *PDGFB* | 190040 | NM_002608.2 | c.670C>T | p.(Arg224Trp) |
| Fam-2 | *BARD1* | 601593 | NM_000465.3 | c.1921C>T *^a^* | p.(Arg641*) |
|  | *RAD50* | 604040 | NM_005732.3 | c.980G>A | p.(Arg327His) |
|  | *SUFU* | 607035 | NM_016169.3 | c.1273G>A | p.(Ala425Thr) |
| Fam-3 | *CHEK2* | 604373 | NM_007194.3 | c.792+2T>C *^a^* | p.? |
|  | *FANCM* | 609644 | NM_020937.2 | c.5204C>T | p.(Ser1735Leu) |
|  | *MN1* | 156100 | NM_002430.2 | c.1632_1637delACAGCA | p.(Gln549_Gln550del) |
| Fam-4 | *ERCC3* | 133510 | NM_000122.1 | c.325C>T *^a^* | p.(Arg109*) |
|  | *FANCM* | 609644 | NM_020937.2 | c.1667A>G | p.(Asp556Gly) |
|  | *FANCM* | 609644 | NM_020937.2 | c.4352A>G | p.(His1451Arg) |
|  | *MSH3* | 600887 | NM_002439.4 | c.2732T>G | p.(Leu911Trp) |
|  | *RNASEL* | 180435 | NM_021133.3 | c.1234C>G | p.(Arg412Gly) |
| Fam-5 | *BARD1* | 601593 | NM_000465.3 | c.2191C>T | p.(Arg731Cys) |
|  | *FANCL* | 608111 | NM_001114636.1 | c.1111_1114dupATTA *^a^* | p.(Thr372Asnfs*13) |
|  | *MN1* | 156100 | NM_002430.2 | c.1574_1575insTCA | p.(Gln524_Gln525insHis) |
| Fam-6 | *ATM* | 607585 | NM_000051.3 | c.1810C>T | p.(Pro604Ser) |
|  | *DDB1* | 600045 | NM_001923.4 | c.2660C>T | p.(Thr887Met) |
|  | *FANCM* | 609644 | NM_020937.2 | c.5791C>T *^a^* | p.(Arg1931*) |
|  | *MEN1* | 613733 | NM_000244.3 | c.1633C>T | p.(Pro545Ser) |
|  | *RHBDF2* | 614404 | NM_024599.5 | c.116A>C | p.(Gln39Pro) |
|  | *TSC2* | 191092 | NM_000548.3 | c.4327G>T | p.(Gly1443Cys) |
| Fam-7 | *BLM* | 604610 | NM_000057.2 | c.3427G>A | p.(Glu1143Lys) |
|  | *MET* | 164860 | NM_001127500.1 | c.504G>T | p.(Glu168Asp) |
|  | *MSH2* | 609309 | NM_000251.2 | c.2785C>T *^a^* | p.(Arg929*) |
|  | *MSH3* | 600887 | NM_002439.4 | c.186_187insGCCGCAGCGCCCGCAGCG | p.(Ala62_Pro63insAlaAlaAlaProAlaAla) |
|  | *PDGFRA* | 173490 | NM_006206.4 | c.661C>T | p.(Leu221Phe) |
|  | *TSC2* | 191092 | NM_000548.3 | c.2712C>G | p.(Phe904Leu) |
| Fam-8 | *APC* | 611731 | NM_000038.4 | c.3920T>A | p.(Ile1307Lys) |
|  | *ATM* | 607585 | NM_000051.3 | c.6084A>T | p.(Gln2028His) |
|  | *DDB2* | 600811 | NM_000107.2 | c.1228G>A | p.(Ala410Thr) |
|  | *ERCC6* | 609413 | NM_000124.2 | c.3122A>C | p.(Gln1041Pro) |
|  | *ERCC6* | 609413 | NM_000124.2 | c.1996C>T | p.(Arg666Cys) |
| Fam-9 | *CHEK2* | 604373 | NM_007194.3 | c.349A>G | p.(Arg117Gly) |
|  | *ERCC6* | 609413 | NM_000124.2 | c.2924G>A | p.(Arg975Gln) |
| Fam-10 | *PMS2* | 600259 | NM_000535.5 | c.1004A>G | p.(Asn335Ser) |
|  | *RNASEL* | 180435 | NM_021133.3 | c.889G>A | p.(Ala297Thr) |
| Fam-11 | *NBN* | 602667 | NM_002485.4 | c.283G>A | p.(Asp95Asn) |
| Fam-12 | *FANCD2* | 613984 | NM_033084.3 | c.172A>G | p.(Ile58Val) |
|  | *RAD50* | 604040 | NM_005732.3 | c.2548C>T | p.(Arg850Cys) |
| Fam-13 | *ATM* | 607585 | NM_000051.3 | c.2289T>A | p.(Phe763Leu) |
|  | *MSH3* | 600887 | NM_002439.4 | c.3339G>A | p.(Met1113Ile) |
|  | *PTCH1* | 601309 | NM_000264.3 | c.113G>A | p.(Gly38Glu) |
|  | *RET* | 164761 | NM_020975.4 | c.166C>A | p.(Leu56Met) |
|  | *SLX4* | 613278 | NM_032444.2 | c.421G>T | p.(Gly141Trp) |
| Fam-14 | *BRCA2* | 600185 | NM_000059.3 | c.7544C>T | p.(Thr2515Ile) |
|  | *MLH3* | 604395 | NM_001040108.1 | c.3315C>A | p.(Asp1105Glu) |
|  | *RHBDF2* | 614404 | NM_024599.5 | c.940G>A | p.(Ala314Thr) |

*^a^* Putative pathogenic variant listed in Table 1.

.

**Table S7. List of variants detected only by WES.** These variants have a high or moderate predicted impact in protein coding transcripts and have a maximum population frequency of 1% (ExAC). Each variant appears once for each protein coding transcript where it has a moderate or high effect.

Due to its length, this table is presented in an excel file.

**Table S8. Expert committee selection of candidate variants from WES analysis of the discovery set.**

| **Family** | **Gene** | **MIM** | **Transcript** | **cDNA** | **Protein** |
| --- | --- | --- | --- | --- | --- |
| Fam-1 | *ATM* | 607585 | NM_000051.3 | c.4776+2_4776+13delTAATAAAAATTT *^a^* | p.? |
|  | *DSC2* | 125645 | NM_024422.4 | c.2686_2687dupGA | p.(Ala897Lysfs*4) |
| Fam-2 | *BARD1* | 601593 | NM_000465.3 | c.1921C>T *^a^* | p.(Arg641*) |
|  | *HPR* | 140210 | NM_020995.3 | c.5+1G>A | p.? |
|  | *MUC16* | 606154 | NM_024690.2 | c.2500_2501delAG | p.(Pro835*) |
| Fam-3 | *CHEK2* | 604373 | NM_007194.3 | c.792+2T>C *^a^* | p.? |
|  | *MAP3K10* | 600137 | NM_002446.3 | c.806G>A | p.(Trp269*) |
| Fam-4 | *ERCC3* | 133510 | NM_000122.1 | c.325C>T *^a^* | p.(Arg109*) |
|  | *MAP4K2* | 603166 | NM_004579.4 | c.89dupT | p.(Tyr31Leufs*37) |
| Fam-5 | *CXCL6* | 138965 | NM_002993.3 | c.239dupT | p.(Val81Glyfs*44) |
|  | *FANCL* | 608111 | NM_001114636.1 | c.1111_1114dupATTA *^a^* | p.(Thr372Asnfs*13) |
| Fam-6 | *FANCM* | 609644 | NM_020937.2 | c.5791C>T *^a^* | p.(Arg1931*) |
|  | *OPN4* | 606665 | NM_001030015.2 | c.403delG | p.(Ala135Profs*35) |
|  | *PARPBP* | 613687 | NM_017915.2 | c.940A>T | p.(Lys314*) |
| Fam-7 | *CTGF* | 121009 | NM_001901.2 | c.546C>A | p.(Tyr182*) |
|  | *EPSTI1* | 607441 | NM_001002264.2 | c.1180_1184dupCCTGA | p.(Glu395Aspfs*24) |
|  | *MSH2* | 609309 | NM_000251.2 | c.2785C>T *^a^* | p.(Arg929*) |
| Fam-8 | *SETD4* | - | NM_017438.2 | c.695delT | p.(Leu232Argfs*4) |
|  | *SMARCA2* | 600014 | NM_001289400.1 | c.3+1G>A | p.? |
| Fam-10 | *ANKRD30A* | 610856 | NM_052997.2 | c.3771_3772delTG | p.(Glu1258Thrfs*28) |
|  | *PLAU* | 191840 | NM_001145031.1 | c.34+2T>C | p.? |
| Fam-11 | *CACNA1G* | 604065 | NM_018896.4 | c.2292C>G | p.(Tyr764*) |
|  | *CARD6* | 609986 | NM_032587.3 | c.88_89delTT | p.(Leu30Argfs*11) |
|  | *UGT2B17* | 601903 | NM_001077.3 | c.311dupA | p.(Asn104Lysfs*19) |
| Fam-12 | *IRAK3* | 604459 | NM_007199.2 | c.317-1G>A | p.? |
|  | *LOXL2* | 606663 | NM_002318.2 | c.1880+1G>A | p.? |
| Fam-13 | *CTNND2* | 604275 | NM_001288717.1 | c.2118+1_2118+2dupTAAA | p.? |
|  | *POLQ* | 604419 | NM_199420.3 | c.4262_4268delTACTATT | p.(Ile1421Argfs*8) |
| Fam-14 | *AURKC* | 603495 | NM_001015878.1 | c.744C>G | p.(Tyr248*) |
|  | *MANF* | 601916 | NM_006010.5 | c.133C>T | p.? |
|  | *RGSL1* | 611012 | NM_001137669.1 | c.1393_1396delATCC | p.(Ile465Profs*22) |

*^a^* Putative pathogenic variant also identified in the panel analysis (listed in Table 1).

**Table S9. Primer sequences for the validations and splicing analyses**

| **Gene** | **Product size (bp)** | **Forward primer** | **Reverse primer** |
| --- | --- | --- | --- |
| *ATM ^a^* | 157 | TGACC[W](http://www.ensembl.org/Homo_sapiens/ZMenu/TextSequence?db=core;factorytype=Location;g=ENSG00000149311;r=11:108222484-108369102;vf=58094881;vf=72272144;vf=72272143)TGTTG[Y](http://www.ensembl.org/Homo_sapiens/ZMenu/TextSequence?db=core;factorytype=Location;g=ENSG00000149311;r=11:108222484-108369102;vf=68624578;vf=31936662)TTTTAAGGATTT | TGCTAGAGCATTACAGATTTTTGAA |
| *ATM* splicing | 400 | GCCAGACAGCCGTGACTTAC | GAAGCTCTCATAATGTCCACCA |
| *BARD1 ^a^* | 195 | AAATTGCTTGATTTTAATGAGAGAGA | TTGTATTAAAAGAAAAATACCAGCTG |
| *CHEK2** | 210 | CAGACAATCACTATCTTTGTTTTTCC | TCTAAGATTATTTTGGGAAGTTATGAA |
| *CHEK2* splicing | 469 | AAACGCCGTCCTTTGAATAA | CAGCCAAGAGCATCTGGTAA |
| *CHEK2* aberrant transcript *^b^* | 245 | GGTGCCTGTGGAGAGGTAAA | AACATTGAGAGCTGGGTCAAA |
| *ERCC3 ^a^* | 193 | CTTGGAAGCCTTCTCTCCAG | AGGGACTCCAGTCTTGCTGAG |
| *FANCL ^a^* | 208 | CTATGACACTATTCTATGACACTATTTC | CTCTGAAGATGATACCAAAATTCC |
| *FANCM ^a^* | 173 | GGAGACACATCAAGGATGTTTAG | GTTGGAACATGAATACCAACATT |
| *MSH2 ^a^* | 170 | AAACAAATGCCCTTTACTGAAA | GCTTATCAATATTACCTTCATTCCA |

*^a^* Primers designed for genomic DNA. *^b^* Primers designed to amplify the aberrant 588-bp transcript.

**SUPPLEMENTARY REFERENCES**

1. De Brakeleer, S. et al. Frequent incidence of BARD1-truncating mutations in germline DNA from triple-negative breast cancer patients*.* *Clin Genet* (2015).

2. Pugh, T. J. et al. The genetic landscape of high-risk neuroblastoma*.* *Nat Genet* **45**, 279-284 (2013).

3. Foley, S. B. et al. Use of Whole Genome Sequencing for Diagnosis and Discovery in the Cancer Genetics Clinic*.* *EBioMedicine* **2**, 74-81 (2015).

4. Akbari, M. R. et al. Mutations in Fanconi anemia genes and the risk of esophageal cancer*.* *Hum Genet* **129**, 573-582 (2011).

5. Peterlongo, P. et al. FANCM c.5791C>T nonsense mutation (rs144567652) induces exon skipping, affects DNA repair activity and is a familial breast cancer risk factor*.* *Hum Mol Genet* **24**, 5345-5355 (2015).

6. Pinto, C. et al. Co-occurrence of nonsense mutations in MSH6 and MSH2 in Lynch syndrome families evidencing that not all truncating mutations are equal*.* *J Hum Genet* **61**, 151-156 (2016).
